# Neuronal Goα and CAPS Regulate Behavioral and Immune Responses to Bacterial Pore-Forming Toxins

**DOI:** 10.1371/journal.pone.0054528

**Published:** 2013-01-17

**Authors:** Ferdinand C. O. Los, Christine Ha, Raffi V. Aroian

**Affiliations:** University of California San Diego, Division of Biological Sciences, Section of cell and developmental biology, La Jolla, California, United States of America; Ecole Polytechnique Federale de Lausanne, Switzerland

## Abstract

Pore-forming toxins (PFTs) are abundant bacterial virulence factors that attack host cell plasma membranes. Host defense mechanisms against PFTs described to date all function in the host tissue that is directly attacked by the PFT. Here we characterize a rapid and fully penetrant cessation of feeding of *Caenorhabditis elegans* in response to PFT attack. We demonstrate via analyses of *C. elegans* mutants that inhibition of feeding by PFT requires the neuronal G protein Goα subunit *goa-1*, and that maintenance of this response requires neuronally expressed calcium activator for protein secretion (CAPS) homolog *unc-31*. Independently from their role in feeding cessation, we find that *goa-1* and *unc-31* are additionally required for immune protection against PFTs. We thus demonstrate that the behavioral and immune responses to bacterial PFT attack involve the cross-talk between the nervous system and the cells directly under attack.

## Introduction

Bacterial infectious diseases rank among the top leading causes of death worldwide. *Mycobacterium tuberculosis, Streptococcus pneumonia* and *Staphylococcus aureus*, among others, are especially problematic because of antibiotic resistance [1]. These, and many other bacterial pathogens, produce pore-forming toxins (PFTs) that contribute significantly to their virulence [2], [3], [4], [5].

Cells possess PFT-defense mechanisms involved in membrane resealing, as well as various molecular defenses [6], [7], [8], [9], [10], [11], [12], [13], [14]. Many genes involved in protection against PFTs were discovered in *C. elegans* using the PFT Cry5B and, where tested, were found to have conserved roles in mammalian cells [7], [11], [14]. All PFT-defense studies published to date involve the cells directly under attack by the PFT. However, as we have previously noted [13], [15], the PFT Cry5B causes an inhibition of feeding behavior in *C. elegans*, suggestive of a neuronal component to PFT responses.

The nervous system can function to fight off pathogens by altering host behavior in response to infection, ranging from classic sickness behavior and emotional responses in humans [16], to pathogen avoidance in *C. elegans* [17], [18], [19]. The neuronal and immune systems are furthermore intimately connected through molecular pathways, likewise observed in humans (*e.g*., neuronal control of cytokine production [20]), as well as in *C. elegans* (*e.g*., neuronal control of antimicrobial peptide expression and noncanonical unfolded protein response genes [21], [22]). Neuronal pathways are involved in *C. elegans*' defense against *Pseudomonas aeruginosa* and fungal infections, controlling behavioral responses [17], [18], [19] as well as downstream molecular defense pathways in the affected tissues [21], [22], [23], [24], [25].

Based on the ease of studying cellular PFT-defenses, innate immunity and the nervous system, we used *C. elegans* to determine whether a neuronal component exists in the defensive responses to a specific class of bacterial virulence factors, PFTs. Here, we report two neuronal pathways that regulate independent behavioral and defensive responses of *C. elegans* to PFTs.

## Results

### PFTs rapidly and reversibly induce feeding cessation

**Figure 1:**
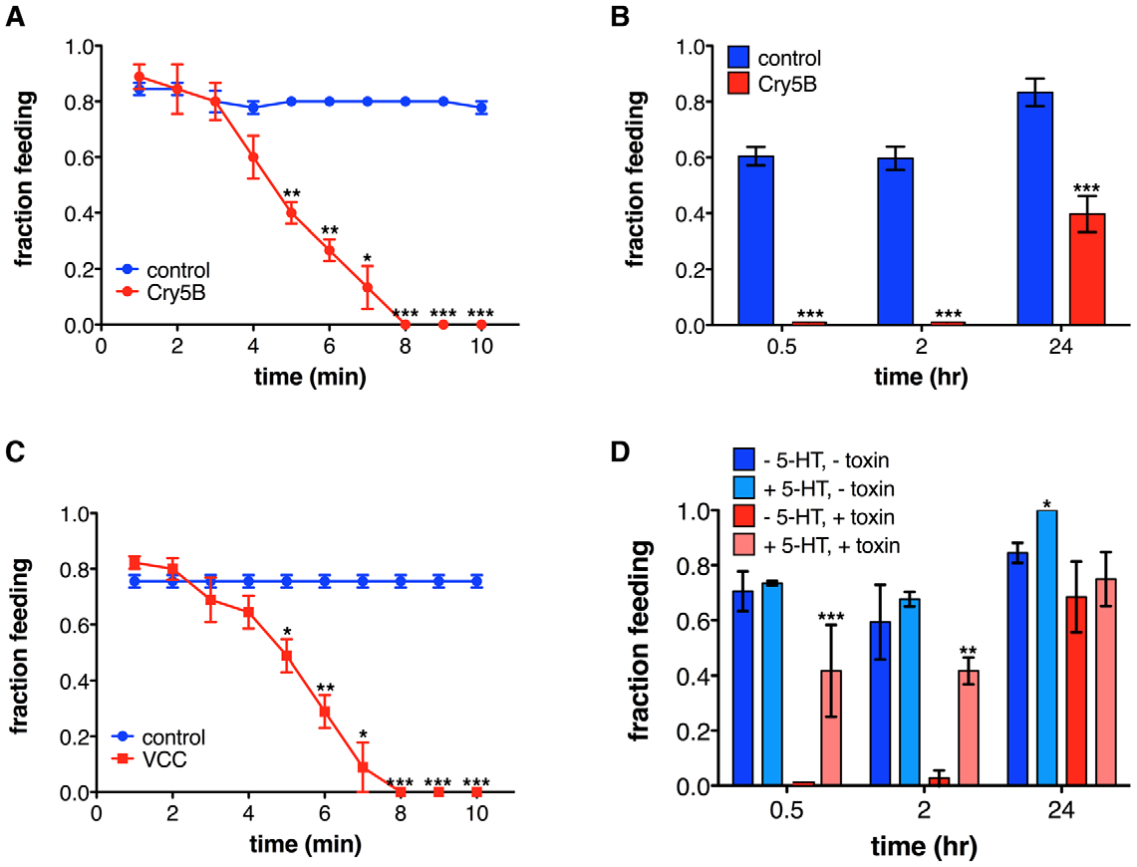
PFTs inhibit feeding in *C. elegans*. (A) *E. coli*-expressed Cry5B rapidly induces cessation of feeding in wild-type *C. elegans*. (Feeding continues normally if animals are transferred to no-Cry5B-control plates instead of Cry5B-expressing plates (Fig. S1B).) (B) 0.5 and 2 hr after transfer to *E. coli*-Cry5B, animals are not feeding, but after 24 hr almost half of the population has resumed. (This is not due to reduced activity of Cry5B (Fig. S1C).) (C) *V. cholerae* expressing VCC induces cessation of feeding, following similar kinetics as Cry5B, whereas *V. cholerae* lacking VCC does not (blue line). (D) Exogenous serotonin causes constitutive feeding on Cry5B. In this and subsequent figures, graphs show mean ± standard error of three experiments unless otherwise described, and statistics indicated are: ns not significant, * p<0.05, ** p<0.01, *** p<0.001. Lack of any symbol indicates no significant difference. Here, statistics indicate significance of difference between PFT and control at the same time point. In all subsequent figures, statistics indicate the difference between mutant and wild type on the same treatment, and where applicable additional statistics are provided in Table S2.

To measure the kinetics of feeding inhibition by PFT, we determined fractions of animals feeding after various exposure times to Cry5B expressed from *Escherichia coli, C. elegans*' normal lab food source. We found that in wild-type animals feeding has ceased in a significant fraction of the population five minutes after transfer to Cry5B, and in the entire population after eight minutes (Fig. 1A). After 30 minutes or two hours animals are still not feeding, but after 24 hours about 40% of the same population has resumed feeding (Fig. 1B). When we exposed animals to *Vibrio cholerae* expressing or lacking their native PFT, *V. cholerae* cytolysin (VCC; a small-pore PFT like Cry5B [26]), we found that it also triggers feeding cessation (Fig. 1C). The same is true for Cry21A, a crystal toxin that belongs to the same family as Cry5B but shares only ∼40% amino acid identity [15] (Fig. S1A). Inhibition of feeding induced by Cry5B, VCC and Cry21A follow very similar kinetics (Fig. 1A, C, S1A), and it thus appears that rapid inhibition of feeding is part of a generalized response of *C. elegans* to small-pore bacterial PFTs.

PFT-induced cessation of feeding can be inhibited by co-treatment with exogenous serotonin, a neurotransmitter known to induce feeding in *C. elegans* [27] (Fig. 1D). Combined with the fact that 40% of animals have resumed feeding after 24 hours (Fig. 1B), this serotonin result indicates that cessation of feeding is reversible and not likely caused by physical damage to the pharynx and suggests that the neural circuitry that controls feeding remains functional in the presence of PFT.

### Goα pathway components are required for cessation of feeding in response to PFTs

Neuronal G-protein signaling pathways control many behaviors in *C. elegans*. Through screening of numerous Goα, Gqα, and Gsα pathway mutants we found a single mutant that shows a penetrant, dramatic loss of feeding inhibition on Cry5B after 30 minutes and two hours, *goa-1(sa734)* (Table S1). *sa734* is a null allele of *goa-1*, the single *C. elegans* homolog of the most abundant G-protein in the mammalian brain, Goα [28], [29]. Goα mediates the signals of many neurotransmitters [29], [30], and in *C. elegans* GOA-1 controls various behaviors such as locomotion, egg-laying, feeding and olfactory adaptation [31], [32], [33].

**Figure 2:**
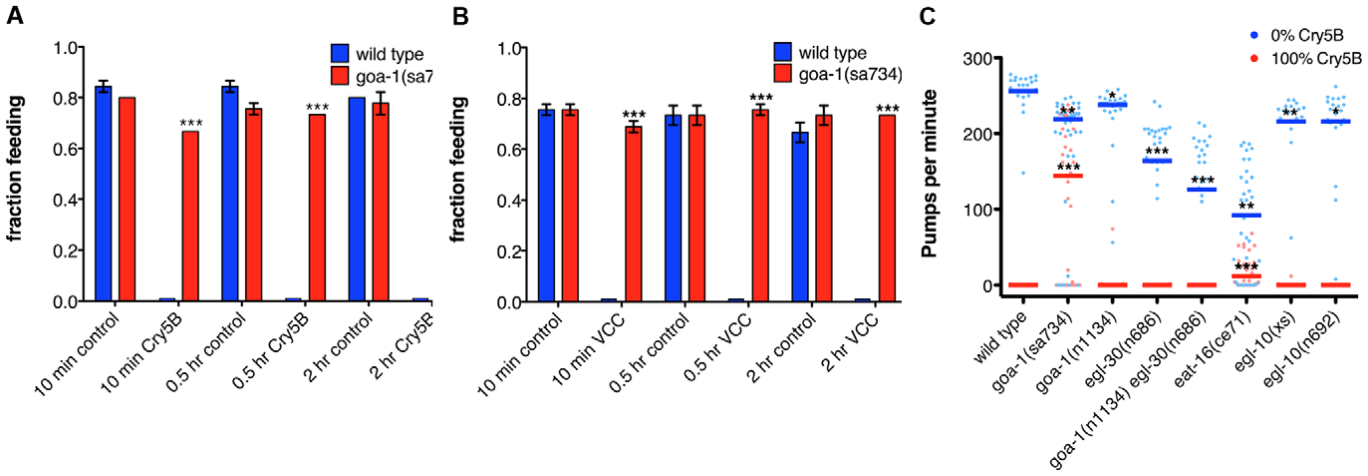
Goα pathway components are required for cessation of feeding in response to PFTs. (A) *goa-1(sa734)* mutant animals constitutively feed on *E. coli*-expressed Cry5B. (B) *goa-1(sa734)* animals constitutively feed on *V. cholerae* expressing VCC. (C) 30 min after transfer to *E. coli*-Cry5B, *goa-1(sa734)* and, to a lesser extend, *eat-16(ce71)* mutant animals have significantly increased feeding rates. (The transfer process itself does not affect feeding rates (Fig. S2C).) Individual measurements of three combined experiments are shown; bars indicate medians. Wild type  =  *C. elegans* N2.

Further analysis of *goa-1(sa734)* feeding behavior shows that after 10, 30 and 120 minutes exposure to Cry5B it has significantly higher fractions of animals feeding than wild type (Fig. 2A). The *goa-1(sa734)* mutation also causes impaired cessation of feeding in response to VCC and Cry21A after 10, 30 and 120 minutes (Fig. 2B, S2A). A different null allele of *goa-1, ep275*, similarly shows significantly increased fractions of animals feeding on Cry5B at all three time points (Fig. S2B), indicating that the phenotype is caused by a loss of *goa-1* function.

Next, we measured pharyngeal pumping rates of several of the Goα and Gqα pathway mutants in absence or presence of Cry5B (The pumping rate is the frequency of muscle contractions in the nematode's posterior pharynx, a measure for its feeding rate [34]). In all strains tested, Cry5B causes a significant reduction of pumping rates after 30 minutes exposure (Fig. 2C, Table S2). However, *goa-1(sa734)* shows significantly higher pumping rates than wild type on Cry5B (Fig. 2C). Interestingly, where complete loss of *goa-1* function results in constitutive pumping on Cry5B, a weak reduction-of-function mutation of *goa-1*, *n1134*, still allows for normal inhibition of feeding by Cry5B (Fig. 2C). *goa-1(n1134)* mutants have a N-terminal truncation of first 4 amino acids causing it to lack the consensus site for myristoylation [33]. They show increased rates of egg laying, an abnormal olfactory response that they share with *goa-1(sa734)*, and a general resistance to phenotypes induced by administration of exogenous serotonin [31], [33]. This may indicate that the reduction of function is too weak to show a phenotype, or that the N-terminal part or proper membrane localization is not required for GOA-1 to inhibit feeding. Thus, Goα, a major pan-neuronal gene in *C. elegans* [32], [33], plays a critical role in regulating PFT-induced inhibition of feeding.

*eat-16(ce71)* carries a loss-of-function mutation in a regulator of G-protein signaling (RGS) protein, functioning downstream of *goa-1* in the control of several behaviors [28], [35], [36]. *eat-16(ce71)* animals show a slightly defective feeding-inhibition response, albeit with less expressivity than that of *goa-1(sa734)* (Fig. 2C).

*egl-30* and *egl-10* are components of the Gq pathway that generally functions antagonistically to the Go pathway [35], [37]. Reduction-of-function or null mutation of *egl-30* or *egl-10* respectively, as expected, did not affect inhibition of feeding by Cry5B (Fig. 2C). However, animals overexpressing *egl-10* (*egl-10(xs)*) or carrying *egl-30 (tg26* and *js126)* gain of function mutations, which share many phenotypes with *goa-1* loss-of-function mutants (*e.g*., scrawny appearance, motility defects) [31], [37], [38], [39], also have a functional PFT-induced feeding-cessation response (Fig. 2C). This indicates that GOA-1 signaling is not sufficiently antagonized in these strains to allow constitutive feeding, or that EGL-30 and EGL-10 are not involved in regulation of pumping behavior in response to PFT.

*unc-31(e928)*, a null allele [40], was also notably different from wild type. These animals initiate the normal feeding-cessation response at 30 minutes PFT exposure, but it is not maintained since by two hours a significant fraction of the animals have resumed feeding (Table S1). Maintenance of feeding cessation is restored when *unc-31* is selectively expressed in neurons (Table S1), indicating it is required in the nervous system to maintain, but not initiate, PFT-induced cessation of feeding. *unc-31* is further discussed below.

### The Goα G-protein pathway is required for PFT defense

**Figure 3:**
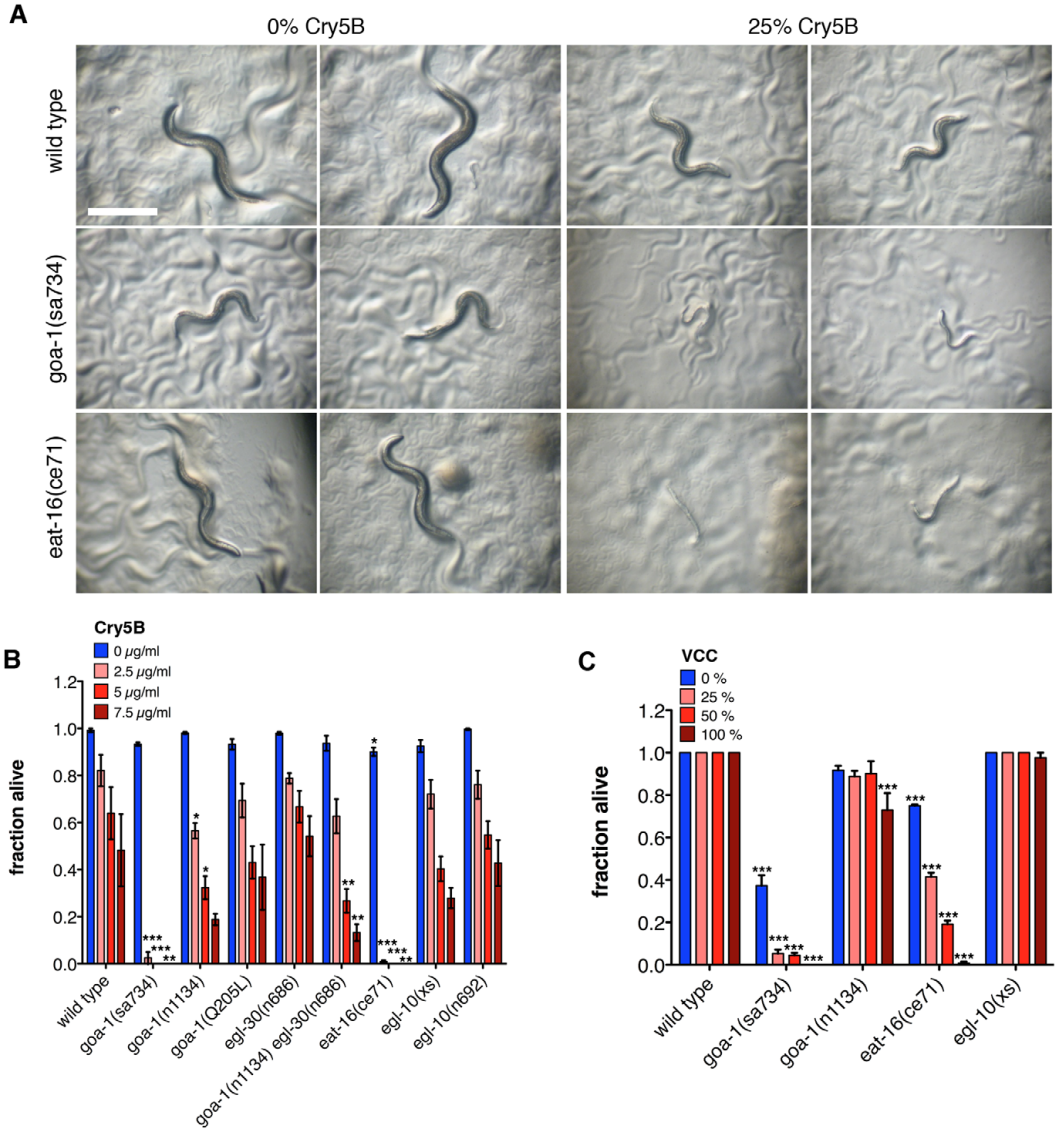
Goα pathway components are required for PFT defense. (A) *goa-1(sa734)* and *eat-16(ce71)* mutants are qualitatively hypersensitive to *E. coli*-expressed Cry5B after 48 hr exposure. 25% Cry5B indicates a 1∶3 dilution of Cry5B-expressing bacteria with non-expressing control bacteria (see Materials and Methods). Scale bar: 500 µm. (B) *goa-1(sa734)* and *eat-16(ce71)* mutants show reduced survival after 8 days on three doses of purified Cry5B. *goa-1(n1134)* and *goa-1(n1134) egl-30(n686)* mutants show hypersensitivity on two Cry5B doses. (C) *V. cholera*e-expressed VCC induces lethality in *goa-1(sa734)* and *eat-16(ce71)* mutants after 24 hr exposure. Percentages VCC indicate fraction of VCC-expressing *V. cholerae* diluted with non-expressing *V. cholerae* (see Materials and Methods).

The mutant strains screened for constitutive pumping in the presence of Cry5B were also examined for qualitatively altered immunity to PFT after 24 and 48 hours exposure to three doses of Cry5B PFT. Mutation of several G-protein signaling pathway genes leads to moderate ***h***ypersensitivity to the ***po***re-forming toxin Cry5B (“Hpo” phenotype [11]), but only loss of *goa-1* or *eat-16* leads to severe Hpo phenotypes at both time points (Table S1, Fig. 3A). The Hpo phenotype is due to loss of *goa-1*, as it is also apparent with the *ep275* null allele (Fig. S3).

For a quantitative analysis of the sensitivities of several Goα and Gqα pathway mutants, we performed dose response assays with purified Cry5B. Consistent with the qualitative assays, *goa-1(sa734)* and *eat-16(ce71)* mutants show significantly decreased survival compared to wild type on the three Cry5B doses tested (Fig. 3B). The *goa-1(n1134)* mild reduction-of-function mutant also shows significant hypersensitivity at two Cry5B doses, although not to the extent of *goa-1(sa734)* and *eat-16(ce71)*. This innate immunity role appears uncoupled from cessation of feeding as *eat-16(ce71)* mutants show only a modest defect of feeding inhibition and *goa-1(n1134)* mutants display a normal feeding-cessation response.

Immunity to Cry5B is unchanged in *egl-30(n686)* animals, and *goa-1(n1134) egl-30(n686)* double mutant animals show decreased Cry5B PFT survival similar to that of *goa-1(n1134)* alone (Fig. 3B). Constitutively active *goa-1(Q205L)* [32] does not affect Cry5B immunity, and neither do loss or overexpression of *egl-10* (Fig. 3B), or *egl-30* gain of function (Table S1). So, in contrast with other phenotypes described in *C. elegans* [31], [35], [36], [41], here the Gqα pathway (EGL-30 and EGL-10) is not antagonistic to the Goα pathway (GOA-1 and EAT-16) and appears not to play a role in Cry5B defense.

Next, we exposed mutants lacking Goα components to *V. cholerae* expressing VCC and scored 24 hours later for survival. *goa-1(sa734)* and *eat-16(ce71)* null mutant animals show significantly decreased survival after 24 hours on various doses of *V. cholerae* expressing VCC compared to a *V. cholerae* strain lacking the PFT, and for *eat-16(ce71)* a dose-response to VCC is evident (Fig. 3C). *V. cholerae* also induces significantly increased lethality in the absence of VCC in both these mutants compared to wild type (Fig. 3C) (further enhanced by the presence of the PFT), which is consistent with the fact that *goa-1(sa734)* animals are hypersensitive to infection by the bacterial pathogen *Pseudomonas aeruginosa* [42]. *goa-1(n1134)* mild reduction-of-function animals show significantly increased lethality on the highest VCC dose only (Fig. 3C). Animals overexpressing *egl-10* show no increased lethality on *V. cholerae*, regardless of the presence or absence of VCC (Fig. 3C). (*egl-30* and *egl-10* reduction-of-function or null mutants could not be tested on *V. cholerae* due to internal hatching of progeny.) Hypersensitivity of these mutants is not likely due to general illness, as in a previous study *goa-1(sa734)* animals showed no hypersensitivity to the heavy metal cadmium, or high osmolarity [42].

### Calcium activator for protein secretion (CAPS) is required in neurons for PFT defense

**Figure 4:**
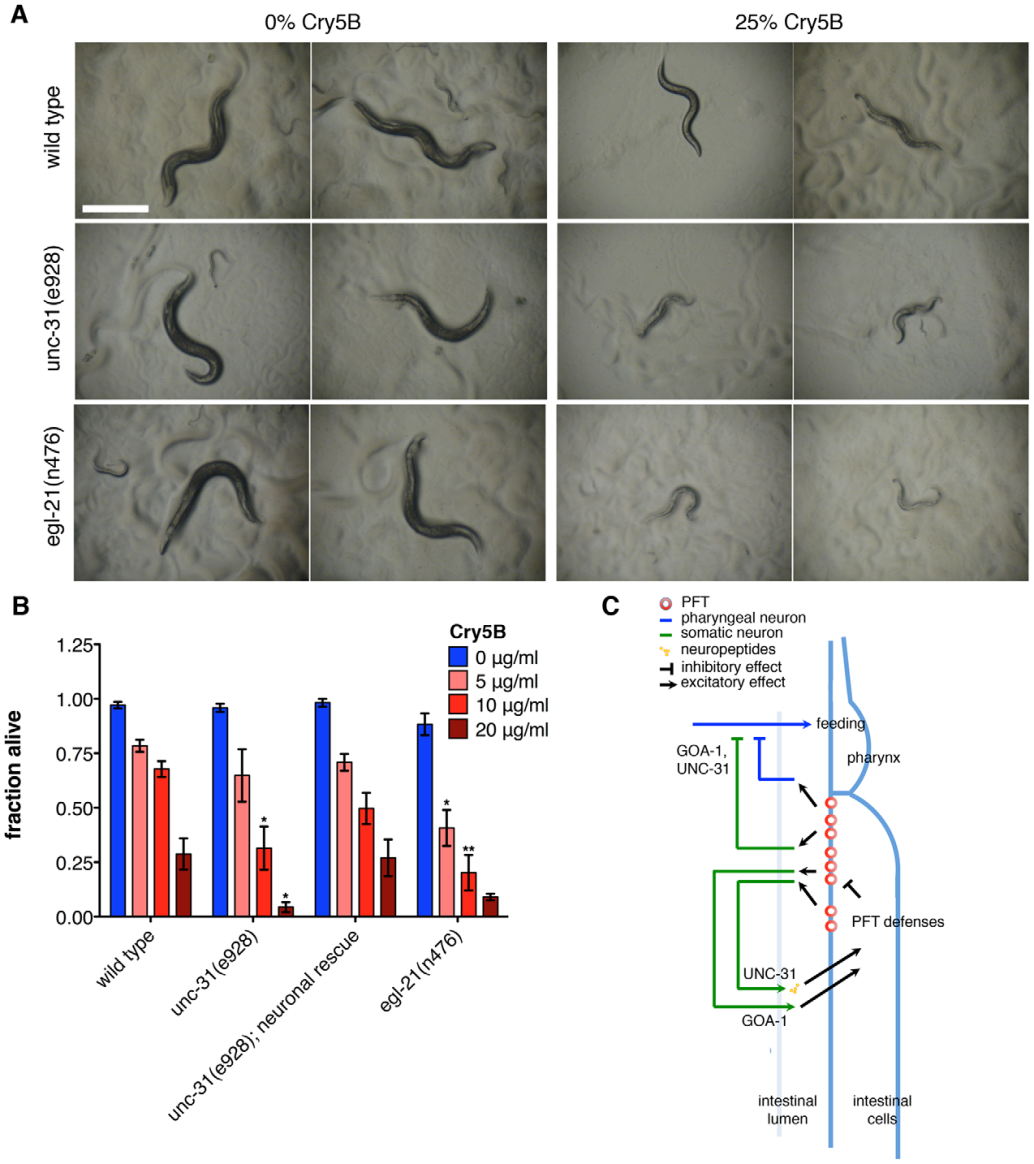
Neuronal CAPS/*unc-31* and *egl-21* function in PFT defense. (A) *unc-31(e928)* and *egl-21(n476)* mutants are qualitatively hypersensitive to *E. coli*-expressed Cry5B after 48 hr exposure. Scale bar: 500 µm. (B) *unc-31(e928)* and *egl-21(n476)* mutants show decreased survival after 8 days on various doses of purified Cry5B respectively. Expression of *unc-31* exclusively in neurons results in wild-type survival. (C) Model outlining the hypothesized roles of GOA-1 and UNC-31 in PFT defense. Cry5B damages the plasma membranes of intestinal cells, resulting in the flux or production of factors that are sensed by neurons. Neuronal signals relayed via GOA-1 and UNC-31 to the pharynx inhibit feeding. GOA-1 and UNC-31 are additionally part of neuronal pathways that activate defenses in the intestine.

*goa-1* mutants are hypersensitive to *P. aeruginosa* infection, which has been linked to hypersecretion in neurons [42]. Consistent with this, calcium activator for protein secretion (CAPS)/*unc-31, egl-3* (protein convertase type 2) and *egl-21* (carboxypeptidase E) mutants, which have reduced neurosecretion because of reduced dense-core vesicle (DCV) exocytosis or defects in DCV contents, are resistant to *P. aeruginosa* infection [42], [43], [44], [45]. To determine if a similar correlation exists for PFT defense, we included *unc-31, egl-3* and *egl-21* mutants in our screen (Table S1). Opposite to the *P. aeruginosa* resistance phenotype, loss of these genes causes hypersensitivity to Cry5B (Table S1, Fig. 4A). In quantitative analyses *unc-31* and *egl-21* mutants show significantly reduced survival on purified Cry5B relative to wild-type controls (Fig. 4B). The *unc-31(e928)* Hpo phenotype is not likely due to general sickness, as loss of *unc-31* increases life span and confers resistance to *P. aeruginosa* [42], [46].

*unc-31* is expressed in all neurons and other secretory cells, but not in the intestine [45]. *unc-31(e928)* animals in which *unc-31* expression was selectively restored to the neurons via the *rab-3* promoter [47] show wild-type sensitivity to Cry5B (Fig. 4B, Table S1). Thus, UNC-31 functions in the neurons to protect *C. elegans* from an attack by PFT on its intestinal cells.

## Discussion

We present data showing that bacterial PFTs rapidly and reversibly inhibit feeding in *C. elegans*, and that the Goα pathway components *goa-1* and downstream *eat-16* are required for this feeding-cessation response. *goa-1* and *eat-16* are furthermore required for defense against PFTs. *egl-21* and neuronal *unc-31* are also required for PFT defense, and neuronal *unc-31*, but not *egl-21*, is required for maintenance of PFT-induced feeding cessation.

As mentioned above, the rapid inhibition of feeding by PFT is not likely caused by damage to the cells or neural circuitry of the pharynx, as PFT-induced feeding inhibition is reversible (Fig. 1B, 1D). Additionally, the pharynx is not directly affected by Cry5B – removing the Cry5B receptor from the intestine but not the pharynx leads to Cry5B resistance and expression of the Cry5B receptor in the intestine but not the pharynx results in Cry5B sensitivity [48], [49]. Cry5B furthermore does not inhibit feeding in intestine-receptor-negative animals (10, 30, 60 and 90 minutes after transfer to Cry5B, 94.9%, 98.2%, 84.7% and 82.0% respectively of *bre-3(ye28)* animals showed normal feeding rates [average of 2 experiments, 23–30 animals each]), and exposure of *C. elegans* to Cry5B results in permeabilization of the intestinal cells but not the pharyngeal cells [13]. These data suggest that rather than the PFT itself, the effects of pore formation in the intestine are sensed and cause a signal to be sent to the pharynx to inhibit pumping. Such a signal could be relayed via synaptic connections or nonsynaptic communication (*e.g*., neuropeptides) between the somatic and pharyngeal nervous systems, or the pharyngeal nervous system could autonomously sense the effects of Cry5B (*e.g*., ion fluxes). Consistent with this model (Fig. 4C), (1) *unc-31* is required in the neurons for a normal feeding-cessation response (Table S1), (2) *goa-1* and *eat-16* are required for a normal feeding-cessation response (Fig. 2A, B, C, S2A, B) and are predominantly (although not exclusively) expressed in neurons [32], [33], and (3) exogenous administration of the neurotransmitter serotonin can suppress the feeding-cessation response (Fig. 1D).

Since loss of *goa-1* results in both continued feeding on Cry5B and increased sensitivity to Cry5B, the two phenotypes could be related–*i.e*., increased consumption of the PFT could cause increased sensitivity because of and increased PFT dose. However, several lines of evidence suggest against such a relationship. Loss of *goa-1* results in hypersensitivity to *V. cholerae* lacking VCC (Fig. 3C). However, wild type animals also continuously feed on *V. cholerae* lacking VCC (Fig. 1C). Furthermore, *goa-1(n1134)* reduction-of-function animals show normal inhibition of feeding (Fig. 2C) but are hypersensitive to Cry5B (Fig. 3B), and a lack of correlation between the expressivities of the feeding-cessation response and PFT immunity was similarly found for the *eat-16(ce71)* mutant (Fig. 2C, 3B). So, although we do not exclude that increased intake of PFT in part contributes to mutant hypersensitivity, we hypothesize that *goa-1* affects feeding inhibition and innate defenses via independent downstream signaling pathways. Such independent roles in behavioral and molecular defensive responses for a single pathway have been shown before in *C. elegans* for the insulin/IGF-I pathway, in the context of a challenge with a pathogenic *Bacillus thuringiensis* strain (likely expressing nematicidal crystal toxins) [50].

The inverse correlation that was found with regard to *P. aeruginosa* resistance for *unc-31* and *goa-1* loss-of-function mutants [42] does not exist for Cry5B PFT defenses (Fig. 3A, B, C, 4A, B). An analogous observation was made for the hypoxia pathway, which when activated protects *C. elegans* against *V. cholerae* expressing VCC but increases its sensitivity to *V. cholerae* lacking VCC [6]. Likewise, UNC-31 activity protects against PFTs but may compromise the resistance to other virulence factors. This finding is likely in accordance with the evolution of the nematode in its natural environment, where different pathogens have evolved different attack strategies that require more complex defensive responses from the nematode.

No direct genetic link was shown between *unc-31* and *goa-1* in *P. aeruginosa* defense [42]. GOA-1 is furthermore thought to be mainly involved in the secretion of synaptic vesicles that contain neurotransmitters such as acetylcholine, whereas UNC-31 is thought to control secretion of dense-core vesicles that contain hormones, serotonin, and neuropeptides such as insulin [47], [51]. *unc-31(e928)* stops feeding after 30 minutes on Cry5B but prematurely resumes feeding at two hours (Table S1), whereas *goa-1(sa734)* is feeding at all time points tested, and to a larger extent than the *unc-31* mutant. In addition, the effects of *goa-1* and *unc-31* mutation on pumping on Cry5B appear to be cumulative, *i.e*., the *goa-1(sa734); unc-31(e928)* double mutant shows higher fractions pumping on Cry5B than either single mutant (Table S1). Therefore, we propose that the PFT-defense functions of *goa-1* and *unc-31* are not coupled to levels of neuronal secretion *per se*, as is hypothesized to be the case for *P. aeruginosa* defense [42], but rather depend on the contents of the different secreted vesicles.

In summary, we show that the *C. elegans* host responses to PFTs include two defense pathways that function outside the tissue under attack, involving the conserved genes *goa-1* and *unc-31*, and that the nematode's nervous system can modulate behavior and immune defenses in response to a specific and highly abundant class of bacterial toxins – PFTs.

## Materials and Methods

### *C. elegans* and bacterial strains

Worm strains used in this study are outlined in Table S3, and were maintained at 20°C on *E. coli* strain OP50, as described [52]. Mutations were confirmed by phenotype where possible, otherwise by PCR band size comparison or DNA sequencing.

Bacterial strains used in this study were *E. coli* OP50, OP50-pQE9, OP50-Cry5B, and OP50-Cry21A, and *V. cholerae* CVD109 and CVD110 [6], [15]. Strains were cultured at 37°C (*E. coli*) or 30°C (*V. cholerae*) in LB broth, supplemented with 50 µg/mL carbenicillin where applicable.

### Feeding assays

To determine fractions of animals in a population that are feeding, *E. coli*-Cry5B, *E. coli*-Cry21A, *V. cholerae*-VCC, and no-PFT control plates were prepared as described [6], [53], [54]. 10–15 L4 animals were transferred to each plate, and the assay was incubated at room temperature and observed after the indicated times. Using a dissecting microscope, any single animal was observed for a maximum of 5–10 seconds, and scored as “feeding” if rhythmical backward movements of the grinder were observed [34]. To determine feeding on 24-hr old plates, plates were prepared as normal, but incubated at 20°C for 24 hr before use. Three independent repeats were performed for each assay.

To quantitatively assess feeding rates of individual animals, we determined pumping rates. Pumping entails the backward movements of the posterior grinder of the pharynx, which can be observed through a dissecting microscope [34]. *E. coli*-Cry5B plates were prepared as above. 10-13 L4 animals were transferred to each plate, and pumping rates were measured for 30 seconds, before transfer, or after 30 minutes incubation at room temperature. Each assay was repeated independently three times. It is of note that *goa-1(n1134)* mutants were reported to have slightly decreased pumping rates [33], which we also observed here for *n1134* and *sa734* (Fig. 2C).

### PFT toxicity assays

For qualitative toxicity assays, including the screening (Table S1), 0%, 10%, 25% and 100% Cry5B plates were used, which are prepared by diluting *E. coli*-Cry5B (100%) bacteria with empty vector control (0%) bacteria at the indicated ratios, as described [53]. To prevent behavioral artifacts due to worms wandering off the toxin [19], [50] bacterial lawns were spread to cover the entire agar plate. 15 L4 animals were transferred to each plate. Assays were incubated at 20°C and observed after 24 and 48 hr. Representative images were taken after 48 hr, using an Olympus SZ60 dissecting microscope linked to a Canon Powershot A620 digital camera, and using Canon Remote Capture software. Three independent repeats were performed for each mutant strain. The same plates prepared for screening Cry5B sensitivity (Table S1) were also used to screen feeding behavior (see below).

Quantitative survival assays with purified Cry5B were performed as described [53]. Cry5B was purified as described [11]. Three independent repeats were performed. Note that toxicity of purified Cry5B on a µg/mL basis sometimes differs from batch to batch, which is why different Cry5B concentrations give similar survival rates for wild-type animals in different experiments (e.g., Fig. 3B versus Fig. 4B). The same batch of Cry5B is used within each complete set of experiments.

VCC survival assays were performed essentially as described [6], except assays were incubated at 20°C, and scored after 24 hr. VCC dilutions were prepared the same way as *E. coli*-Cry5B dilutions. Animals that showed internal hatching of progeny (“bagging”) or the intestine protruding through the vulva (“exploding”) were censored. Pilot assays involving the use of 5-fluoro-2′-deoxy-uridine (FUdR) to prevent the development (and internal hatching) of progeny revealed that FUdR causes *V. cholerae* to lose its pathogenicity. Therefore, *egl-30*-like strains could not be tested here, as without inhibiting the development of their progeny the terminal phenotype of most of these animals is bagging due to their low egg-laying rates. An *E. coli* OP50 control was included with each assay, which showed no significant lethality for any of the strains after 24 hr (data not shown). Three independent repeats were performed with 30–50 animals per treatment. After 24 hours, wild-type animals are qualitatively affected by the presence of VCC but show no lethality on any of the VCC doses (Fig. 3C). VCC-induced lethality is noticeable in wild-type animals at later time points [6].

To circumvent known issues with findings being behavioral artifacts due to worms wandering off the toxin [19], [25], [50], all assays involving agar plates (Fig. 1A–D, 2A–C, 3A, C, 4A, S1A–C, S2A–C, S3) were performed using “full lawns” of bacteria [19], [25]. Use of liquid assays (Fig. 3B, 4B), and expression of Cry5B from OP50 (the maintenance food source) make interference by avoidance behavior less likely as well.

### Statistical analyses

For Fig. 1A–C, and S1A–C t-tests were used to compare each pair of measurements. For Fig. 1D, 2A, B, 3B, C, 4B and S2A, B one-way ANOVA with Dunnett's comparison of means was performed for each treatment. For Fig. 2C and S2C a Wilcoxon test was performed. For Table S1 fractions feeding on Cry5B were normalized to fractions feeding on control plates at each time point and one-way ANOVA of these normalized values was performed. Means of the mutants were compared to mean of wild type using a Dunnett's test.

Statistics were calculated using JMP 9.0 software (SAS Institute). Prism 5.0 Software (GraphPad) was used to draw graphs. p values, means or medians and confidence intervals or interquartile ranges of all experiments performed for this study are provided in Table S2.

## Supporting Information

Figure S1 **Cry21A inhibits feeding, transfer does not alter feeding, and Cry5B plates retain potency over 24** **hr.** (A) Animals transferred to *E. coli* expressing Cry21A rapidly stop feeding, whereas animals transferred to control plates do not. (B) Fractions of wild-type animals feeding at various time points after transfer are the same as before transfer. (C) Wild-type animals transferred to 24-hr old *E. coli*-Cry5B plates are not feeding 10 or 30 min after transfer. Statistics indicate difference between 24-hr old control and 24-hr old Cry5B plate. Here and in all subsequent supplemental figures graphs show mean ± standard error of 3 experiments, and statistics indicated are: ns not significant, * p<0.05, ** p<0.01, *** p<0.001. Additional statistics are provided in Table S2.
(TIF)

Figure S2 ***goa-1*** **null mutants constitutively feed on Cry21A and Cry5B, and transferring worms does not affect their pumping rates.** (A) *E. coli*-expressed Cry21A inhibits feeding in wild-type animals after the indicated exposure times, but does not inhibit feeding in *goa-1(sa734)* mutants. (B) *goa-1(ep275)* constitutively feeds on *E.* coli-expressed Cry5B. (C) 30 minutes after transfer to plates with *E. coli* not expressing PFT, pumping rates are the same as before transfer. Bars show mean ± standard error of 3 experiments, and dots are individual measurements of all three experiments. Additional statistics are provided in Table S2.
(TIF)

Figure S3 **Goα is required for PFT defense.** After 48 hr, *goa-1(ep275)* mutants are qualitatively hypersensitive to *E. coli*-expressed Cry5B. Scale bar  = 500 µm.
(TIF)

Table S1 **Feeding and sensitivity phenotypes of mutants on Cry5B.** Includes References S1 for Table S1.
(DOCX)

Table S2 **Statistical analyses.**
(DOCX)

Table S3 ***C. elegans*** **strains used in this study.**
(DOCX)
