# Supplementary figures and images for: Neuronal Goα and CAPS Regulate Behavioral and Immune Responses to Bacterial Pore-Forming Toxins
Source: PLoS One. 2013 Jan 17;8(1):e54528. doi: 10.1371/journal.pone.0054528 (PMC3547950; doi:10.1371/journal.pone.0054528)

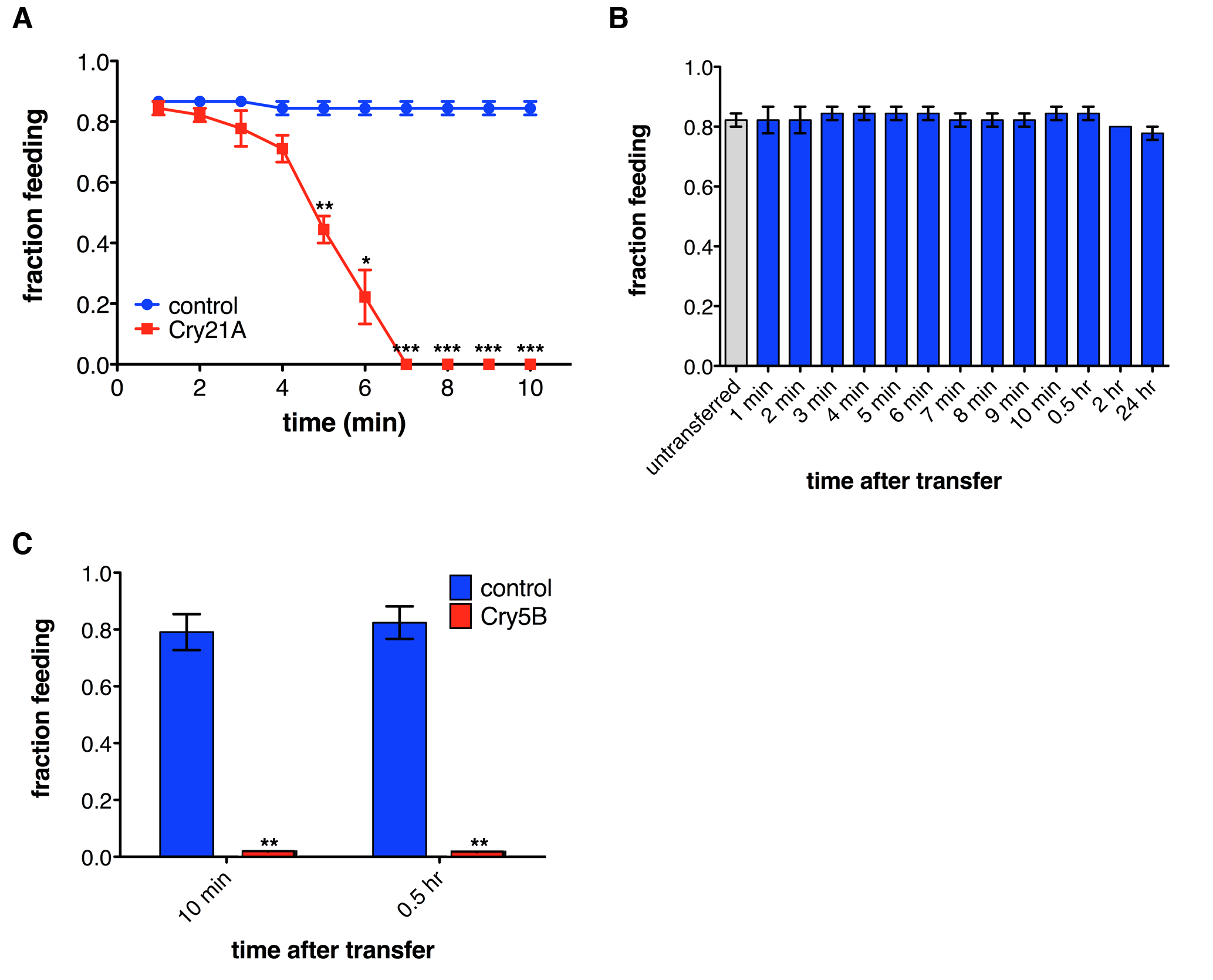

Supplement: Figure S1 — Cry21A inhibits feeding, transfer does not alter feeding, and Cry5B plates retain potency over 24 hr. (A) Animals transferred to E. coli expressing Cry21A rapidly stop feeding, whereas animals transferred to control plates do not. (B) Fractions of wild-type animals feeding at various time points after transfer are the same as before transfer. (C) Wild-type animals transferred to 24-hr old E. coli-Cry5B plates are not feeding 10 or 30 min after transfer. Statistics indicate difference between 24-hr old control and 24-hr old Cry5B plate. Here and in all subsequent supplemental figures graphs show mean ± standard error of 3 experiments, and statistics indicated are: ns not significant, * p<0.05, ** p<0.01, *** p<0.001. Additional statistics are provided in Table S2. (TIF) [file pone.0054528.s001.tif]

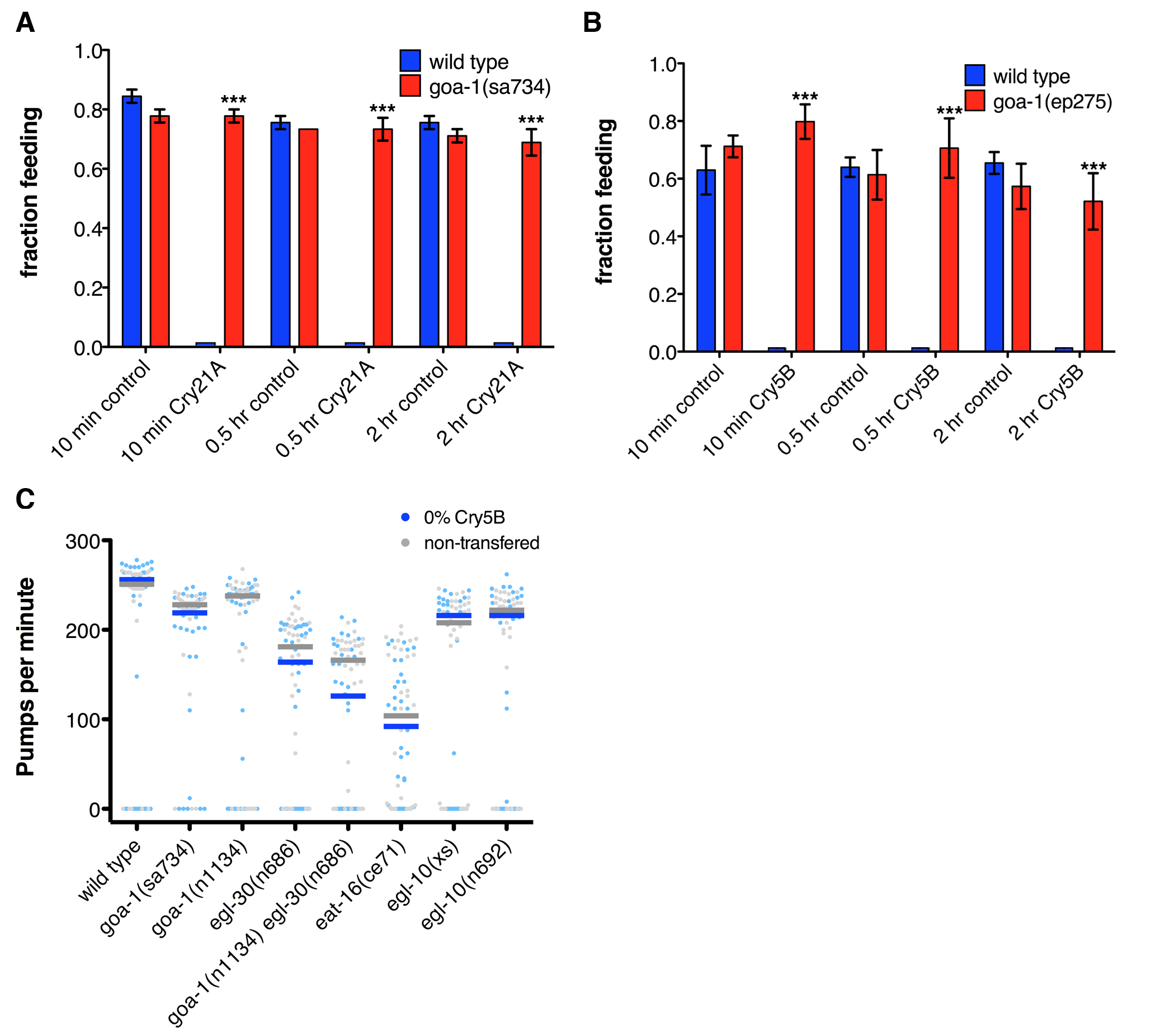

Supplement: Figure S2 — goa-1 null mutants constitutively feed on Cry21A and Cry5B, and transferring worms does not affect their pumping rates. (A) E. coli-expressed Cry21A inhibits feeding in wild-type animals after the indicated exposure times, but does not inhibit feeding in goa-1(sa734) mutants. (B) goa-1(ep275) constitutively feeds on E. coli-expressed Cry5B. (C) 30 minutes after transfer to plates with E. coli not expressing PFT, pumping rates are the same as before transfer. Bars show mean ± standard error of 3 experiments, and dots are individual measurements of all three experiments. Additional statistics are provided in Table S2. (TIF) [file pone.0054528.s002.tif]

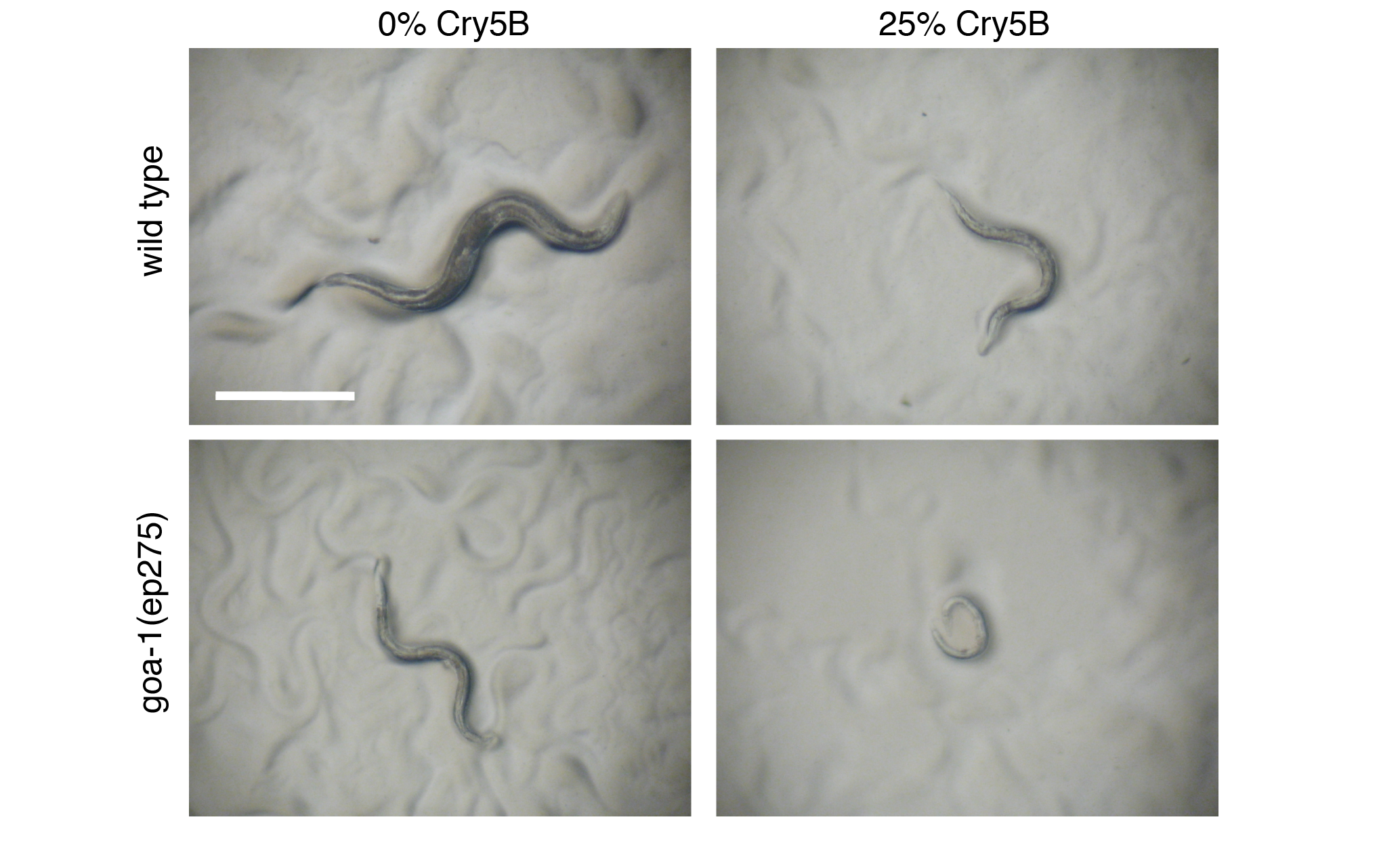

Supplement: Figure S3 — Goα is required for PFT defense. After 48 hr, goa-1(ep275) mutants are qualitatively hypersensitive to E. coli-expressed Cry5B. Scale bar = 500 µm. (TIF) [file pone.0054528.s003.tif]
