# Supplementary material for: Neuronal Goα and CAPS Regulate Behavioral and Immune Responses to Bacterial Pore-Forming Toxins
Source: PLoS One. 2013 Jan 17;8(1):e54528. doi: 10.1371/journal.pone.0054528 (PMC3547950; doi:10.1371/journal.pone.0054528)
Supplement: Table S1 — Feeding and sensitivity phenotypes of mutants on Cry5B. Includes References S1 for Table S1. (DOCX) [file pone.0054528.s004.docx]

**Table S1. Feeding and sensitivity phenotypes of mutants on Cry5B**

| genotype | mutation type^1^ | effect of mutation | fraction pumping after 30 min^2^ | | | | | | fraction pumping after 2 hr^2^ | | | | | | Cry5B sensitivity^3^ | | reference |
| --- | --- | --- | --- | --- | --- | --- | --- | --- | --- | --- | --- | --- | --- | --- | --- | --- | --- |
|  |  |  | control | | | Cry5B | | | control | | | Cry5B | | | 24 hr | 48 hr |  |
| wild type | - | - | 0.60 | ± | 0.03 | 0.00 | ± | 0.00 | 0.60 | ± | 0.04 | 0.01 | ± | 0.01 | wt | wt | [[1](#_ENREF_1)] |
| *gsa-1(ce94)* | GF | activates Gs | 0.58 | ± | 0.09 | 0.12 | ± | 0.08 | 0.54 | ± | 0.06 | 0.07 | ± | 0.03 | wt | wt-mHpo | [[2](#_ENREF_2)] |
| *acy-1 (md1756)* | GF | activates Gs | 0.41 | ± | 0.12 | 0.03 | ± | 0.03 | 0.39 | ± | 0.06 | 0.03 | ± | 0.03 | wt | wt | [[2](#_ENREF_2),[3](#_ENREF_3)] |
| *pde-4(ce268)* | RF/LF | increased cAMP levels | 0.52 | ± | 0.12 | 0.03 | ± | 0.03 | 0.56 | ± | 0.04 | 0.03 | ± | 0.03 | wt-mHpo | wt | [[4](#_ENREF_4)] |
| *kin-2(ce179)* | RF | activates Gs | 0.82 | ± | 0.10 | 0.06 | ± | 0.03 | 0.37 | ± | 0.07 | 0.12 | ± | 0.06 | wt-mHpo | mHpo | [[2](#_ENREF_2)] |
| *goa-1(sa734)* | LF | reduces Go | 0.57 | ± | 0.04 | 0.53 | ± | 0.03 | 0.60 | ± | 0.00 | 0.53 | ± | 0.07 | Hpo | Hpo | [[5](#_ENREF_5),[6](#_ENREF_6),[7](#_ENREF_7)] |
| *goa-1 (n1134)* | RF | reduces Go | 0.56 | ± | 0.15 | 0.06 | ± | 0.03 | 0.53 | ± | 0.07 | 0.00 | ± | 0.00 | wt | mHpo | [[8](#_ENREF_8)] |
| *egl-30(n686)* | RF | reduces Gq | 0.42 | ± | 0.10 | 0.00 | ± | 0.00 | 0.60 | ± | 0.06 | 0.00 | ± | 0.00 | wt | wt-mHpo | [[5](#_ENREF_5),[9](#_ENREF_9)] |
| *egl-30(tg26)* | GF | increases Gq | 0.09 | ± | 0.05 | 0.03 | ± | 0.03 | 0.22 | ± | 0.12 | 0.00 | ± | 0.00 | wt | wt-mHpo | [[5](#_ENREF_5)] |
| *egl-30(js126)* | GF | increases Gq | 0.09 | ± | 0.05 | 0.00 | ± | 0.00 | 0.19 | ± | 0.19 | 0.00 | ± | 0.00 | wt | mHpo | [[10](#_ENREF_10)] |
| *goa-1 (n1134)*  *egl-30(n686)* | RF RF | reduces Go and Gq | 0.43 | ± | 0.06 | 0.00 | ± | 0.00 | 0.52 | ± | 0.09 | 0.03 | ± | 0.03 | wt-mHpo | mHpo | [[8](#_ENREF_8)] |
| *eat-16(ce71)* | LF | reduces Go / reduces Gq inhibition | 0.49 | ± | 0.07 | 0.15 | ± | 0.04 | 0.63 | ± | 0.09 | 0.13 | ± | 0.09 | Hpo | Hpo | [[7](#_ENREF_7),[10](#_ENREF_10),[11](#_ENREF_11)] |
| *egl-10(n692)* | LF | decreases Goα inhibition | 0.34 | ± | 0.05 | 0.00 | ± | 0.00 | 0.31 | ± | 0.03 | 0.00 | ± | 0.00 | wt | wt-mHpo | [[11](#_ENREF_11),[12](#_ENREF_12),[13](#_ENREF_13)] |
| *egl-10(xs)* | XS | increases Goα inhibition | 0.71 | ± | 0.02 | 0.00 | ± | 0.00 | 0.45 | ± | 0.05 | 0.12 | ± | 0.06 | wt-mHpo | mHpo | [[7](#_ENREF_7),[10](#_ENREF_10),[14](#_ENREF_14),[15](#_ENREF_15)] |
| *ric-8 (md1909)* | RF | reduces Gq and potentially Gs | 0.56 | ± | 0.16 | 0.00 | ± | 0.00 | 0.46 | ± | 0.08 | 0.03 | ± | 0.03 | wt | mHpo | [[16](#_ENREF_16)] |
| *dgk-1(nu62)* | LF | increases DAG levels | 0.63 | ± | 0.13 | 0.03 | ± | 0.03 | 0.55 | ± | 0.13 | 0.18 | ± | 0.01 | wt-mHpo | mHpo | [[12](#_ENREF_12),[17](#_ENREF_17)] |
| *egl-8(nu88)* | LF | reduces DAG levels | 0.43 | ± | 0.27 | 0.00 | ± | 0.00 | 0.35 | ± | 0.09 | 0.04 | ± | 0.04 | mHpo | mHpo | [[12](#_ENREF_12),[18](#_ENREF_18),[19](#_ENREF_19)] |
| *unc-13(e51)* | LF | reduced neurotransmitter release | 0.52 | ± | 0.15 | 0.11 | ± | 0.03 | 0.74 | ± | 0.18 | 0.03 | ± | 0.03 | wt | wt | [[18](#_ENREF_18),[20](#_ENREF_20)] |
| *tph-1 (mg280)* | LF | reduces serotonin levels | 0.49 | ± | 0.07 | 0.00 | ± | 0.00 | 0.42 | ± | 0.06 | 0.00 | ± | 0.00 | wt | wt | [[21](#_ENREF_21),[22](#_ENREF_22)] |
| *unc-31(e928)* | LF | reduces neuropeptide release and Gs | 0.75 | ± | 0.13 | 0.06 | ± | 0.03 | 0.77 | ± | 0.04 | 0.27 | ± | 0.03 | mHpo | mHpo | [[5](#_ENREF_5),[23](#_ENREF_23),[24](#_ENREF_24)] |
| *unc-31 (e928); ceEx117* | LF;neuro-nal rescue | *UNC-31* only functional in neurons | 0.68 | ± | 0.09 | 0.03 | ± | 0.03 | 0.67 | ± | 0.13 | 0.03 | ± | 0.03 | wt-mHpo | wt-mHpo | [[25](#_ENREF_25)] |
| *egl-21(n476)* | LF/RF | neuropeptide defective | 0.48 | ± | 0.10 | 0.06 | ± | 0.03 | 0.49 | ± | 0.18 | 0.00 | ± | 0.00 | wt | mHpo | [[25](#_ENREF_25),[26](#_ENREF_26)] |
| *egl-3(gk238)* | LF | neuropeptide defective | 0.58 | ± | 0.05 | 0.08 | ± | 0.08 | 0.47 | ± | 0.20 | 0.06 | ± | 0.06 | wt-mHpo | Hpo | [[26](#_ENREF_26),[27](#_ENREF_27)] |
| *goa-1 (sa734);*  *unc-31(e928)* | LF;LF | reduces Go and neuropeptide release and Gs | 0.77 | ± | 0.07 | 0.72 | ± | 0.10 | 0.53 | ± | 0.10 | 0.66 | ± | 0.09 | mHpo-Hpo | Hpo | [[5](#_ENREF_5),[28](#_ENREF_28)] |

^1^LF = loss of function (null), RF = reduction of function (non-null), GF = gain of function, XS = overexpression.

^2^Average of at least three independent experiments ± standard error.

^3^Qualitative interpretation based on at least three independent experiments, with two doses of Cry5B each. wt = wild type sensitivity, Hpo = severely hypersensitive, mHpo = mildly hypersensitive.

^4^*egl-10(xs)* is strain MT8190 (see Table S3). *egl-10* is overexpressed in this strain using 4,000 base pairs of 5’ flanking sequence, and we therefore assume localization of its expression is the same as for native *egl-10* [[14](#_ENREF_14)].

Statistical analysis showed two mutants to be significantly different at both time points, *goa-1(sa734)* and *goa-1(sa734);unc-31(e928)* (p<0.001 for both mutants at both time points). No other mutants showed a significant difference at either time point.

**References S1 for Table S1**

1. Brenner S (1974) The genetics of *Caenorhabditis elegans*. Genetics 77: 71-94.

2. Schade MA, Reynolds NK, Dollins CM, Miller KG (2005) Mutations that rescue the paralysis of *Caenorhabditis elegans* ric-8 (synembryn) mutants activate the G alpha(s) pathway and define a third major branch of the synaptic signaling network. Genetics 169: 631-649.

3. Reynolds NK, Schade MA, Miller KG (2005) Convergent, RIC-8-dependent Galpha signaling pathways in the *Caenorhabditis elegans* synaptic signaling network. Genetics 169: 651-670.

4. Charlie NK, Thomure AM, Schade MA, Miller KG (2006) The Dunce cAMP phosphodiesterase PDE-4 negatively regulates G alpha(s)-dependent and G alpha(s)-independent cAMP pools in the *Caenorhabditis elegans* synaptic signaling network. Genetics 173: 111-130.

5. Charlie NK, Schade MA, Thomure AM, Miller KG (2006) Presynaptic UNC-31 (CAPS) is required to activate the G alpha(s) pathway of the *Caenorhabditis elegans* synaptic signaling network. Genetics 172: 943-961.

6. Robatzek M, Thomas JH (2000) Calcium/calmodulin-dependent protein kinase II regulates *Caenorhabditis elegans* locomotion in concert with a G(o)/G(q) signaling network. Genetics 156: 1069-1082.

7. van Swinderen B, Metz LB, Shebester LD, Mendel JE, Sternberg PW, et al. (2001) Goalpha regulates volatile anesthetic action in *Caenorhabditis elegans*. Genetics 158: 643-655.

8. Shyn SI, Kerr R, Schafer WR (2003) Serotonin and Go modulate functional states of neurons and muscles controlling *C. elegans* egg-laying behavior. Curr Biol 13: 1910-1915.

9. Wang Q, Wadsworth WG (2002) The C domain of netrin UNC-6 silences calcium/calmodulin-dependent protein kinase- and diacylglycerol-dependent axon branching in Caenorhabditis elegans. The Journal of neuroscience : the official journal of the Society for Neuroscience 22: 2274-2282.

10. Matsuki M, Kunitomo H, Iino Y (2006) Goalpha regulates olfactory adaptation by antagonizing Gqalpha-DAG signaling in *Caenorhabditis elegans*. Proc Natl Acad Sci U S A 103: 1112-1117.

11. van der Linden AM, Simmer F, Cuppen E, Plasterk RH (2001) The G-protein beta-subunit GPB-2 in *Caenorhabditis elegans* regulates the G(o)alpha-G(q)alpha signaling network through interactions with the regulator of G-protein signaling proteins EGL-10 and EAT-16. Genetics 158: 221-235.

12. Locke CJ, Kautu BB, Berry KP, Lee SK, Caldwell KA, et al. (2009) Pharmacogenetic analysis reveals a post-developmental role for Rac GTPases in *Caenorhabditis elegans* GABAergic neurotransmission. Genetics 183: 1357-1372.

13. Miller KG, Alfonso A, Nguyen M, Crowell JA, Johnson CD, et al. (1996) A genetic selection for *Caenorhabditis elegans* synaptic transmission mutants. Proceedings of the National Academy of Sciences of the United States of America 93: 12593-12598.

14. Koelle MR, Horvitz HR (1996) EGL-10 regulates G protein signaling in the *C. elegans* nervous system and shares a conserved domain with many mammalian proteins. Cell 84: 115-125.

15. Miller KG, Emerson MD, Rand JB (1999) Goalpha and diacylglycerol kinase negatively regulate the Gqalpha pathway in *C. elegans*. Neuron 24: 323-333.

16. Miller KG, Emerson MD, McManus JR, Rand JB (2000) RIC-8 (Synembryn): a novel conserved protein that is required for G(q)alpha signaling in the *C. elegans* nervous system. Neuron 27: 289-299.

17. Jose AM, Koelle MR (2005) Domains, amino acid residues, and new isoforms of *Caenorhabditis elegans* diacylglycerol kinase 1 (DGK-1) important for terminating diacylglycerol signaling in vivo. The Journal of biological chemistry 280: 2730-2736.

18. Tokuoka SM, Saiardi A, Nurrish SJ (2008) The mood stabilizer valproate inhibits both inositol- and diacylglycerol-signaling pathways in Caenorhabditis elegans. Molecular biology of the cell 19: 2241-2250.

19. Yook K, Hodgkin J (2007) Mos1 mutagenesis reveals a diversity of mechanisms affecting response of *Caenorhabditis elegans* to the bacterial pathogen Microbacterium nematophilum. Genetics 175: 681-697.

20. Richmond JE, Davis WS, Jorgensen EM (1999) UNC-13 is required for synaptic vesicle fusion in *C. elegans*. Nat Neurosci 2: 959-964.

21. Hobson RJ, Hapiak VM, Xiao H, Buehrer KL, Komuniecki PR, et al. (2006) SER-7, a *Caenorhabditis elegans* 5-HT7-like receptor, is essential for the 5-HT stimulation of pharyngeal pumping and egg laying. Genetics 172: 159-169.

22. Sze JY, Victor M, Loer C, Shi Y, Ruvkun G (2000) Food and metabolic signalling defects in a *Caenorhabditis elegans* serotonin-synthesis mutant. Nature 403: 560-564.

23. Ailion M, Inoue T, Weaver CI, Holdcraft RW, Thomas JH (1999) Neurosecretory control of aging in *Caenorhabditis elegans*. Proc Natl Acad Sci U S A 96: 7394-7397.

24. Daniels SA, Ailion M, Thomas JH, Sengupta P (2000) egl-4 acts through a transforming growth factor-beta/SMAD pathway in *Caenorhabditis elegans* to regulate multiple neuronal circuits in response to sensory cues. Genetics 156: 123-141.

25. Kawli T, Tan MW (2008) Neuroendocrine signals modulate the innate immunity of *Caenorhabditis elegans* through insulin signaling. Nat Immunol 9: 1415-1424.

26. Jacob TC, Kaplan JM (2003) The EGL-21 carboxypeptidase E facilitates acetylcholine release at *Caenorhabditis elegans* neuromuscular junctions. J Neurosci 23: 2122-2130.

27. Lee BH, Ashrafi K (2008) A TRPV channel modulates *C. elegans* neurosecretion, larval starvation survival, and adult lifespan. PLoS genetics 4: e1000213.

28. Edwards SL, Charlie NK, Richmond JE, Hegermann J, Eimer S, et al. (2009) Impaired dense core vesicle maturation in Caenorhabditis elegans mutants lacking Rab2. The Journal of cell biology 186: 881-895.
