# Supplementary material for: Neuronal Goα and CAPS Regulate Behavioral and Immune Responses to Bacterial Pore-Forming Toxins
Source: PLoS One. 2013 Jan 17;8(1):e54528. doi: 10.1371/journal.pone.0054528 (PMC3547950; doi:10.1371/journal.pone.0054528)
Supplement: Table S2 — Statistical analyses. (DOCX) [file pone.0054528.s005.docx]

**Table S2. Statistical analyses**

| **condition A^1^** | **condition B^1^** | **mean A^1^** | **S.E.M. A^1^** | **mean B^1^** | **S.E.M. B^1^** | **dife-rence^2^** | **lower CL^3^** | **upper CL^3^** | **p-value^3^** | **sum-mary^4^** |
| --- | --- | --- | --- | --- | --- | --- | --- | --- | --- | --- |
| *Fig. 1A - fractions feeding after transfer to Cry5B* | | | | | | | | | | |
| 1min, control | 1min, Cry5B | 0.844 | 0.022 | 0.889 | 0.044 | 0.044 | -0.116 | 0.204 | 0.4328 | ns |
| 2min, control | 2min, Cry5B | 0.844 | 0.022 | 0.844 | 0.089 | 0.000 | -0.355 | 0.355 | 1.0000 | ns |
| 3min, control | 3min, Cry5B | 0.800 | 0.038 | 0.800 | 0.067 | 0.000 | -0.237 | 0.237 | 1.0000 | ns |
| 4min, control | 4min, Cry5B | 0.778 | 0.022 | 0.600 | 0.077 | 0.178 | -0.124 | 0.480 | 0.1385 | ns |
| 5min, control | 5min, Cry5B | 0.800 | 0.000 | 0.400 | 0.038 | 0.400 | 0.234 | 0.566 | 0.0091 | ** |
| 6min, control | 6min, Cry5B | 0.800 | 0.000 | 0.267 | 0.038 | 0.533 | 0.368 | 0.699 | 0.0052 | ** |
| 7min, control | 7min, Cry5B | 0.800 | 0.000 | 0.133 | 0.077 | 0.667 | 0.335 | 0.998 | 0.0131 | * |
| 8min, control | 8min, Cry5B | 0.800 | 0.000 | 0.000 | 0.000 | 0.800 | . | . | . | *** |
| 9min, control | 9min, Cry5B | 0.800 | 0.000 | 0.000 | 0.000 | 0.800 | . | . | . | *** |
| 10min, control | 10min, Cry5B | 0.778 | 0.022 | 0.000 | 0.000 | 0.778 | 0.682 | 0.873 | 0.0008 | *** |
| *Fig. 1B - fractions feeding after transfer to Cry5B* | | | | | | | | | | |
| 0.5h, control | 0.5h, Cry5B | 0.604 | 0.033 | 0.000 | 0.000 | 0.604 | 0.524 | 0.684 | <0.0001 | *** |
| 2h, control | 2h, Cry5B | 0.597 | 0.042 | 0.000 | 0.000 | 0.597 | 0.499 | 0.695 | <0.0001 | *** |
| 24h, control | 24h, Cry5B | 0.854 | 0.048 | 0.398 | 0.065 | 0.457 | 0.283 | 0.630 | <0.0001 | *** |
| *Fig. 1C - fractions feeding after transfer to VCC* | | | | | | | | | | |
| 1min, control | 1min, VCC | 0.756 | 0.022 | 0.822 | 0.022 | 0.067 | -0.021 | 0.154 | 0.1012 | ns |
| 2min, control | 2min, VCC | 0.756 | 0.022 | 0.800 | 0.038 | 0.044 | -0.092 | 0.181 | 0.3868 | ns |
| 3min, control | 3min, VCC | 0.756 | 0.022 | 0.689 | 0.080 | 0.067 | -0.249 | 0.383 | 0.4970 | ns |
| 4min, control | 4min, VCC | 0.756 | 0.022 | 0.644 | 0.059 | 0.111 | -0.110 | 0.332 | 0.1908 | ns |
| 5min, control | 5min, VCC | 0.756 | 0.022 | 0.489 | 0.059 | 0.267 | 0.046 | 0.488 | 0.0327 | * |
| 6min, control | 6min, VCC | 0.756 | 0.022 | 0.289 | 0.059 | 0.467 | 0.246 | 0.688 | 0.0085 | ** |
| 7min, control | 7min, VCC | 0.756 | 0.022 | 0.089 | 0.089 | 0.667 | 0.312 | 1.022 | 0.0132 | * |
| 8min, control | 8min, VCC | 0.756 | 0.022 | 0.000 | 0.000 | 0.756 | 0.660 | 0.851 | 0.0009 | *** |
| 9min, control | 9min, VCC | 0.756 | 0.022 | 0.000 | 0.000 | 0.756 | 0.660 | 0.851 | 0.0009 | *** |
| 10min, control | 10min, VCC | 0.756 | 0.022 | 0.000 | 0.000 | 0.756 | 0.660 | 0.851 | 0.0009 | *** |
| *Fig. 1D - fractions feeding after transfer to Cry5B, in presence of serotonin* | | | | | | | | | | |
| 0.5h, -Cry5B, -5-HT | 0.5h, -Cry5B, +5-HT | 0.706 | 0.072 | 0.735 | 0.008 | 0.029 |  |  | 0.7072 | ns |
| 2h, -Cry5B, -5-HT | 2h, -Cry5B, +5-HT | 0.593 | 0.136 | 0.677 | 0.267 | 0.083 |  |  | 0.5792 | ns |
| 24h, -Cry5B, -5-HT | 24h, -Cry5B, +5-HT | 0.845 | 0.036 | 1.000 | 0.000 | 0.155 |  |  | 0.0129 | * |
| 0.5h, +Cry5B, -5H-T | 0.5h, +Cry5B, +5-HT | 0.000 | 0.000 | 0.583 | 0.000 | 0.583 |  |  | <0.0001 | *** |
| 2h, +Cry5B,-5-HT | 2h, +Cry5B, +5-HT | 0.028 | 0.028 | 0.417 | 0.048 | 0.389 |  |  | 0.0022 | ** |
| 24h, +Cry5B, -5-HT | 24h, +Cry5B, +5-HT | 0.685 | 0.129 | 0.750 | 0.098 | 0.065 |  |  | 0.7100 | ns |
| 0.5h, -Cry5B, -5-HT | 0.5h, +Cry5B, -5H-T | 0.706 | 0.072 | 0.000 | 0.000 | 0.706 |  |  | 0.0006 | *** |
| 2h, -Cry5B, -5-HT | 2h, +Cry5B,-5-HT | 0.593 | 0.136 | 0.028 | 0.028 | 0.566 |  |  | 0.0150 | * |
| 24h, -Cry5B, -5-HT | 24h, +Cry5B, -5-HT | 0.845 | 0.036 | 0.685 | 0.129 | 0.160 |  |  | 0.2976 | ns |
| 0.5h, -Cry5B, +5-HT | 0.5h, +Cry5B, +5-HT | 0.735 | 0.008 | 0.583 | 0.000 | 0.152 |  |  | 0.0006 | *** |
| 2h, -Cry5B, +5-HT | 2h, +Cry5B, +5-HT | 0.677 | 0.027 | 0.417 | 0.048 | 0.260 |  |  | 0.0091 | ** |
| 24h, -Cry5B, +5-HT | 24h, +Cry5B, +5-HT | 1.000 | 0.000 | 0.750 | 0.098 | 0.250 |  |  | 0.0636 | ns |
| *Fig. 2A - fractions feeding after transfer to Cry5B* | | | | | | | | | | |
| wild type, control, 10min | wild type, Cry5B, 10min | 0.844 | 0.022 | 0.000 | 0.000 | 0.844 |  |  | <0.0001 | *** |
| *goa-1(sa734)*, control, 10min | *goa-1(sa734)*, Cry5B, 10min | 0.800 | 0.000 | 0.667 | 0.000 | 0.133 |  |  | . | . |
| wild type, control, 10min | *goa-1(sa734)*, control, 10min | 0.844 | 0.022 | 0.800 | 0.000 | 0.044 |  |  | 0.1161 | ns |
| wild type, Cry5B, 10min | *goa-1(sa734)*, Cry5B, 10min | 0.000 | 0.000 | 0.667 | 0.000 | 0.667 |  |  | <0.0001 | *** |
| wild type, control, 0.5hr | wild type, Cry5B, 0.5hr | 0.844 | 0.022 | 0.000 | 0.000 | 0.844 |  |  | <0.0001 | *** |
| *goa-1(sa734)*, control, 0.5hr | *goa-1(sa734)*, Cry5B, 0.5hr | 0.756 | 0.022 | 0.733 | 0.000 | 0.022 |  |  | 0.3739 | ns |
| wild type, control, 0.5hr | *goa-1(sa734)*, control, 0.5hr | 0.844 | 0.022 | 0.756 | 0.022 | 0.089 |  |  | 0.0474 | * |
| wild type, Cry5B, 0.5hr | *goa-1(sa734)*, Cry5B, 0.5hr | 0.000 | 0.000 | 0.733 | 0.000 | 0.733 |  |  | <0.0001 | *** |
| wild type, control, 2hr | wild type, Cry5B, 2hr | 0.800 | 0.000 | 0.000 | 0.000 | 0.800 |  |  | . | . |
| *goa-1(sa734)*, control, 2hr | *goa-1(sa734)*, Cry5B, 2hr | 0.778 | 0.044 | 0.667 | 0.000 | 0.111 |  |  | 0.0668 | ns |
| wild type, control, 2hr | *goa-1(sa734)*, control, 2hr | 0.800 | 0.000 | 0.778 | 0.044 | 0.022 |  |  | 0.6433 | ns |
| wild type, Cry5B, 2hr | *goa-1(sa734)*, Cry5B, 2hr | 0.000 | 0.000 | 0.667 | 0.000 | 0.667 |  |  | <0.0001 | *** |
| *Fig. 2B - fractions feeding after transfer to VCC* | | | | | | | | | | |
| wild type, control, 10min | wild type, VCC, 10min | 0.756 | 0.022 | 0.000 | 0.000 | 0.756 |  |  | <0.0001 | *** |
| *goa-1(sa734)*, control, 10min | *goa-1(sa734)*, VCC, 10min | 0.756 | 0.022 | 0.689 | 0.022 | 0.067 |  |  | 0.1012 | ns |
| wild type, control, 10min | *goa-1(sa734)*, control, 10min | 0.756 | 0.022 | 0.756 | 0.022 | 0.000 |  |  | 1.0000 | ns |
| wild type, VCC, 10min | *goa-1(sa734)*, VCC, 10min | 0.000 | 0.000 | 0.689 | 0.022 | 0.689 |  |  | <0.0001 | *** |
| wild type, control, 0.5hr | wild type, VCC, 0.5hr | 0.733 | 0.038 | 0.000 | 0.000 | 0.733 |  |  | <0.0001 | *** |
| *goa-1(sa734)*, control, 0.5hr | *goa-1(sa734)*, VCC, 0.5hr | 0.733 | 0.038 | 0.756 | 0.022 | 0.022 |  |  | 0.6433 | ns |
| wild type, control, 0.5hr | *goa-1(sa734)*, control, 0.5hr | 0.733 | 0.038 | 0.733 | 0.038 | 0.000 |  |  | 1.0000 | ns |
| wild type, VCC, 0.5hr | *goa-1(sa734)*, VCC, 0.5hr | 0.756 | 0.022 | 0.000 | 0.000 | 0.756 |  |  | <0.0001 | *** |
| wild type, control, 2hr | wild type, VCC, 2hr | 0.667 | 0.038 | 0.000 | 0.000 | 0.667 |  |  | <0.0001 | *** |
| *goa-1(sa734)*, control, 2hr | *goa-1(sa734)*, VCC, 2hr | 0.733 | 0.038 | 0.733 | 0.000 | 0.000 |  |  | 1.0000 | ns |
| wild type, control, 2hr | *goa-1(sa734)*, control, 2hr | 0.667 | 0.038 | 0.733 | 0.038 | 0.067 |  |  | 0.2879 | ns |
| wild type, VCC, 2hr | *goa-1(sa734)*, VCC, 2hr | 0.000 | 0.000 | 0.733 | 0.000 | 0.733 |  |  | <0.0001 | *** |
| *Fig. 2C - pumping rates after transfer to Cry5B* | | | | | | | | | | |
| wild type, control | wild type, Cry5B | 256 | 0-269.5 | 0 | 0-0 | 256 |  |  | <0.0001 | *** |
| *goa-1(n1134) egl-30(n686)*, control | *goa-1(n1134) egl-30(n686)*, Cry5B | 126 | 0-178 | 0 | 0-0 | 126 |  |  | <0.0001 | *** |
| *goa-1(sa734)*, control | *goa-1(sa734)*, Cry5B | 219 | 170-229.5 | 144 | 0-206 | 75 |  |  | 0.0013 | ** |
| *eat-16(ce71)*, control | *eat-16(ce71)*, Cry5B | 92 | 3-146 | 12 | 0-30.5 | 80 |  |  | 0.0005 | *** |
| *egl-30(n686)*, control | *egl-30(n686)*, Cry5B | 164 | 0-203 | 0 | 0-0 | 164 |  |  | <0.0001 | *** |
| *egl-10(n692)*, control | *egl-10(n692)*, Cry5B | 216 | 0-238 | 0 | 0-0 | 216 |  |  | <0.0001 | *** |
| *goa-1(n1134)*, control | *goa-1(n1134)*, Cry5B | 237 | 69.5-243.5 | 0 | 0-0 | 237 |  |  | <0.0001 | *** |
| *egl-10(xs)*, control | *egl-10(xs)*, Cry5B | 216 | 0-232 | 0 | 0-0 | 216 |  |  | <0.0001 | *** |
| wild type, control | *goa-1(n1134) egl-30(n686)*, control | 256 | 0-269.5 | 126 | 0-178 | 130 |  |  | 0.0002 | *** |
| wild type, control | *goa-1(sa734)*, control | 256 | 0-269.5 | 219 | 170-229.5 | 37 |  |  | 0.0084 | ** |
| wild type, control | *eat-16(ce71)*, control | 256 | 0-269.5 | 92 | 3-146 | 164 |  |  | 0.0015 | ** |
| wild type, control | *egl-30(n686)*, control | 256 | 0-269.5 | 164 | 0-203 | 92 |  |  | 0.0008 | *** |
| wild type, control | *egl-10(n692)*, control | 256 | 0-269.5 | 216 | 0-238 | 40 |  |  | 0.0051 | ** |
| wild type, control | *goa-1(n1134)*, control | 256 | 0-269.5 | 237 | 69.5-243.5 | 19 |  |  | 0.0196 | * |
| wild type, control | *egl-10(xs)*, control | 256 | 0-269.5 | 216 | 0-232 | 40 |  |  | 0.0017 | ** |
| wild type, Cry5B | *goa-1(n1134) egl-30(n686)*, Cry5B | 0 | 0-0 | 0 | 0-0 | 0 |  |  | 0.3324 | ns |
| wild type, Cry5B | *goa-1(sa734)*, Cry5B | 0 | 0-0 | 144 | 0-206 | 144 |  |  | <0.0001 | *** |
| wild type, Cry5B | *eat-16(ce71)*, Cry5B | 0 | 0-0 | 12 | 0-30.5 | 12 |  |  | <0.0001 | *** |
| wild type, Cry5B | *egl-30(n686)*, Cry5B | 0 | 0-0 | 0 | 0-0 | 0 |  |  | 0.9825 | ns |
| wild type, Cry5B | *egl-10(n692)*, Cry5B | 0 | 0-0 | 0 | 0-0 | 0 |  |  | 0.3324 | ns |
| wild type, Cry5B | *goa-1(n1134)*, Cry5B | 0 | 0-0 | 0 | 0-0 | 0 |  |  | 0.4902 | ns |
| wild type, Cry5B | *egl-10(xs)*, Cry5B | 0 | 0-0 | 0.000 | 0-0 | 0 |  |  | 0.5458 | ns |
| *Fig. 3B - survival rates after 8 days on Cry5B* | | | | | | | | | | |
| wild type, 0µg/ml Cry5B | wild type, 2.5µg/ml Cry5B | 0.992 | 0.008 | 0.821 | 0.067 | 0.171 |  |  | 0.5216 | ns |
| wild type, 0µg/ml Cry5B | wild type, 5µg/ml Cry5B | 0.992 | 0.008 | 0.640 | 0.111 | 0.352 |  |  | 0.0913 | ns |
| wild type, 0µg/ml Cry5B | wild type, 7.5µg/ml Cry5B | 0.992 | 0.008 | 0.482 | 0.153 | 0.510 |  |  | 0.0179 | * |
| wild type, 0µg/ml Cry5B | *egl-10(n692)*, 0µg/ml Cry5B | 0.992 | 0.008 | 0.925 | 0.026 | 0.067 |  |  | 0.0752 | ns |
| wild type, 0µg/ml Cry5B | *goa-1(n1134) egl-30(n686)*, 0µg/ml Cry5B | 0.992 | 0.008 | 0.937 | 0.032 | 0.055 |  |  | 0.1866 | ns |
| wild type, 0µg/ml Cry5B | *egl-30(n686)*, 0µg/ml Cry5B | 0.992 | 0.008 | 0.933 | 0.022 | 0.059 |  |  | 0.1395 | ns |
| wild type, 0µg/ml Cry5B | *goa-1(sa734)*, 0µg/ml Cry5B | 0.992 | 0.008 | 0.933 | 0.008 | 0.059 |  |  | 0.1443 | ns |
| wild type, 0µg/ml Cry5B | *egl-10(xs)*, 0µg/ml Cry5B | 0.992 | 0.008 | 0.997 | 0.003 | 0.005 |  |  | 1.0000 | ns |
| wild type, 0µg/ml Cry5B | *goa-1(gf)*, 0µg/ml Cry5B | 0.992 | 0.008 | 0.979 | 0.006 | 0.012 |  |  | 0.9973 | ns |
| wild type, 0µg/ml Cry5B | *eat-16(ce71)*, 0µg/ml Cry5B | 0.992 | 0.008 | 0.901 | 0.018 | 0.091 |  |  | 0.0103 | * |
| wild type, 0µg/ml Cry5B | *goa-1(n1134)*, 0µg/ml Cry5B | 0.992 | 0.008 | 0.981 | 0.005 | 0.011 |  |  | 0.9989 | ns |
| wild type, 2.5µg/ml Cry5B | *egl-10(n692)*, 2.5µg/ml Cry5B | 0.821 | 0.067 | 0.721 | 0.061 | 0.101 |  |  | 0.6509 | ns |
| wild type, 2.5µg/ml Cry5B | *goa-1(n1134) egl-30(n686)*, 2.5µg/ml Cry5B | 0.821 | 0.067 | 0.627 | 0.072 | 0.194 |  |  | 0.0900 | ns |
| wild type, 2.5µg/ml Cry5B | *egl-30(n686)*, 2.5µg/ml Cry5B | 0.821 | 0.067 | 0.693 | 0.072 | 0.128 |  |  | 0.4127 | ns |
| wild type, 2.5µg/ml Cry5B | *goa-1(sa734)*, 2.5µg/ml Cry5B | 0.821 | 0.067 | 0.025 | 0.025 | 0.796 |  |  | <0.0001 | *** |
| wild type, 2.5µg/ml Cry5B | *egl-10(xs)*, 2.5µg/ml Cry5B | 0.821 | 0.067 | 0.761 | 0.060 | 0.061 |  |  | 0.9493 | ns |
| wild type, 2.5µg/ml Cry5B | *goa-1(gf)*, 2.5µg/ml Cry5B | 0.821 | 0.067 | 0.788 | 0.022 | 0.033 |  |  | 0.9986 | ns |
| wild type, 2.5µg/ml Cry5B | *eat-16(ce71)*, 2.5µg/ml Cry5B | 0.821 | 0.067 | 0.010 | 0.005 | 0.811 |  |  | <0.0001 | *** |
| wild type, 2.5µg/ml Cry5B | *goa-1(n1134)*, 2.5µg/ml Cry5B | 0.821 | 0.067 | 0.565 | 0.033 | 0.256 |  |  | 0.0162 | * |
| wild type, 5µg/ml Cry5B | *egl-10(n692)*, 5µg/ml Cry5B | 0.640 | 0.111 | 0.404 | 0.052 | 0.236 |  |  | 0.0692 | ns |
| wild type, 5µg/ml Cry5B | *goa-1(n1134) egl-30(n686)*, 5µg/ml Cry5B | 0.640 | 0.111 | 0.267 | 0.051 | 0.373 |  |  | 0.0024 | ** |
| wild type, 5µg/ml Cry5B | *egl-30(n686)*, 5µg/ml Cry5B | 0.640 | 0.111 | 0.430 | 0.069 | 0.209 |  |  | 0.1259 | ns |
| wild type, 5µg/ml Cry5B | *goa-1(sa734)*, 5µg/ml Cry5B | 0.640 | 0.111 | 0.000 | 0.000 | 0.640 |  |  | <0.0001 | *** |
| wild type, 5µg/ml Cry5B | *egl-10(xs)*, 5µg/ml Cry5B | 0.640 | 0.111 | 0.547 | 0.059 | 0.092 |  |  | 0.8334 | ns |
| wild type, 5µg/ml Cry5B | *goa-1(gf)*, 5µg/ml Cry5B | 0.640 | 0.111 | 0.667 | 0.067 | 0.028 |  |  | 0.9999 | ns |
| wild type, 5µg/ml Cry5B | *eat-16(ce71)*, 5µg/ml Cry5B | 0.640 | 0.111 | 0.000 | 0.000 | 0.640 |  |  | <0.0001 | *** |
| wild type, 5µg/ml Cry5B | *goa-1(n1134)*, 5µg/ml Cry5B | 0.640 | 0.111 | 0.323 | 0.049 | 0.317 |  |  | 0.0100 | * |
| wild type, 7.5µg/ml Cry5B | *egl-10(n692)*, 7.5µg/ml Cry5B | 0.482 | 0.153 | 0.279 | 0.043 | 0.203 |  |  | 0.4241 | ns |
| wild type, 7.5µg/ml Cry5B | *goa-1(n1134) egl-30(n686)*, 7.5µg/ml Cry5B | 0.482 | 0.153 | 0.133 | 0.036 | 0.350 |  |  | 0.0486 | * |
| wild type, 7.5µg/ml Cry5B | *egl-30(n686)*, 7.5µg/ml Cry5B | 0.482 | 0.153 | 0.368 | 0.139 | 0.115 |  |  | 0.8942 | ns |
| wild type, 7.5µg/ml Cry5B | *goa-1(sa734)*, 7.5µg/ml Cry5B | 0.482 | 0.153 | 0.000 | 0.000 | 0.482 |  |  | 0.0046 | ** |
| wild type, 7.5µg/ml Cry5B | *egl-10(xs)*, 7.5µg/ml Cry5B | 0.482 | 0.153 | 0.428 | 0.097 | 0.054 |  |  | 0.9984 | ns |
| wild type, 7.5µg/ml Cry5B | *goa-1(gf)*, 7.5µg/ml Cry5B | 0.482 | 0.153 | 0.542 | 0.085 | 0.060 |  |  | 0.9970 | ns |
| wild type, 7.5µg/ml Cry5B | *eat-16(ce71)*, 7.5µg/ml Cry5B | 0.482 | 0.153 | 0.000 | 0.000 | 0.482 |  |  | 0.0046 | ** |
| wild type, 7.5µg/ml Cry5B | *goa-1(n1134)*, 7.5µg/ml Cry5B | 0.482 | 0.153 | 0.188 | 0.024 | 0.294 |  |  | 0.1202 | ns |
| *Fig. 3C - survival rates after 24 hr on VCC* | | | | | | | | | | |
| wild type, 0% VCC | wild type, 25% VCC | 1.000 | 0.000 | 1.000 | 0.000 | 0.000 | -0.146 | 0.146 | 1.0000 | ns |
| wild type, 0% VCC | wild type, 50% VCC | 1.000 | 0.000 | 1.000 | 0.000 | 0.000 | -0.146 | 0.146 | . | ns |
| wild type, 0% VCC | wild type, 100% VCC | 1.000 | 0.000 | 1.000 | 0.000 | 0.000 | -0.146 | 0.146 | . | ns |
| wild type, 0% VCC | *goa-1(sa734)*, 0% VCC | 1.000 | 0.000 | 0.373 | 0.049 | 0.627 | 0.480 | 0.773 | <.0001 | *** |
| wild type, 0% VCC | *eat-16(ce71)*, 0% VCC | 1.000 | 0.000 | 0.750 | 0.006 | 0.250 | 0.104 | 0.396 | <.0001 | *** |
| wild type, 0% VCC | *goa-1(n1134)*, 0% VCC | 1.000 | 0.000 | 0.917 | 0.021 | 0.083 | -0.063 | 0.229 | 0.8142 | ns |
| wild type, 0% VCC | *egl-10(xs)*, 0% VCC | 1.000 | 0.000 | 1.000 | 0.000 | 0.000 | -0.146 | 0.146 | . | ns |
| wild type, 25% VCC | *goa-1(sa734)*, 25% VCC | 1.000 | 0.000 | 0.053 | 0.018 | 0.947 | 0.800 | 1.093 | <.0001 | *** |
| wild type, 25% VCC | *eat-16(ce71)*, 25% VCC | 1.000 | 0.000 | 0.415 | 0.019 | 0.585 | 0.439 | 0.731 | <.0001 | *** |
| wild type, 25% VCC | *goa-1(n1134)*, 25% VCC | 1.000 | 0.000 | 0.888 | 0.026 | 0.112 | -0.034 | 0.258 | 0.3353 | ns |
| wild type, 25% VCC | *egl-10(xs)*, 25% VCC | 1.000 | 0.000 | 1.000 | 0.000 | 0.000 | -0.146 | 0.146 | 1.0000 | ns |
| wild type, 50% VCC | *goa-1(sa734)*, 50% VCC | 1.000 | 0.000 | 0.045 | 0.011 | 0.955 | 0.809 | 1.102 | <.0001 | *** |
| wild type, 50% VCC | *eat-16(ce71)*, 50% VCC | 1.000 | 0.000 | 0.192 | 0.017 | 0.808 | 0.662 | 0.955 | <.0001 | *** |
| wild type, 50% VCC | *goa-1(n1134)*, 50% VCC | 1.000 | 0.000 | 0.901 | 0.059 | 0.099 | -0.048 | 0.245 | 0.5556 | ns |
| wild type, 50% VCC | *egl-10(xs)*, 50% VCC | 1.000 | 0.000 | 1.000 | 0.000 | 0.000 | -0.146 | 0.146 | . | ns |
| wild type, 100% VCC | *goa-1(sa734)*, 100% VCC | 1.000 | 0.000 | 0.000 | 0.000 | 1.000 | 0.854 | 1.146 | <.0001 | *** |
| wild type, 100% VCC | *eat-16(ce71)*, 100% VCC | 1.000 | 0.000 | 0.007 | 0.007 | 0.993 | 0.846 | 1.139 | <.0001 | *** |
| wild type, 100% VCC | *goa-1(n1134)*, 100% VCC | 1.000 | 0.000 | 0.730 | 0.079 | 0.270 | 0.124 | 0.417 | <.0001 | *** |
| wild type, 100% VCC | *egl-10(xs)*, 100% VCC | 1.000 | 0.000 | 0.977 | 0.023 | 0.023 | -0.123 | 0.170 | 1.0000 | ns |
| *goa-1(sa734)*, 0% VCC | *goa-1(sa734)*, 25% VCC | 0.373 | 0.049 | 0.053 | 0.018 | 0.320 | 0.174 | 0.467 | <.0001 | *** |
| *goa-1(sa734)*, 0% VCC | *goa-1(sa734)*, 50% VCC | 0.373 | 0.049 | 0.045 | 0.011 | 0.329 | 0.182 | 0.475 | <.0001 | *** |
| *goa-1(sa734)*, 0% VCC | *goa-1(sa734)*, 100% VCC | 0.373 | 0.049 | 0.000 | 0.000 | 0.373 | 0.227 | 0.520 | <.0001 | *** |
| *eat-16(ce71)*, 0% VCC | *eat-16(ce71)*, 25% VCC | 0.750 | 0.006 | 0.415 | 0.019 | 0.335 | 0.189 | 0.481 | <.0001 | *** |
| *eat-16(ce71)*, 0% VCC | *eat-16(ce71)*, 50% VCC | 0.750 | 0.006 | 0.192 | 0.017 | 0.558 | 0.412 | 0.705 | <.0001 | *** |
| *eat-16(ce71)*, 0% VCC | *eat-16(ce71)*, 100% VCC | 0.750 | 0.006 | 0.007 | 0.007 | 0.743 | 0.596 | 0.889 | <.0001 | *** |
| *Fig. 4B - survival rates after 8 days on Cry5B* | | | | | | | | | | |
| wild type, 0µg/ml Cry5B | wild type, 5µg/ml Cry5B | 0.972 | 0.015 | 0.784 | 0.028 | 0.187 |  |  | 0.0377 | * |
| wild type, 0µg/ml Cry5B | wild type, 10µg/ml Cry5B | 0.972 | 0.015 | 0.678 | 0.036 | 0.294 |  |  | 0.0034 | ** |
| wild type, 0µg/ml Cry5B | wild type, 20µg/ml Cry5B | 0.972 | 0.015 | 0.288 | 0.072 | 0.684 |  |  | <.0001 | *** |
| wild type, 0µg/ml Cry5B | *unc-31(e928)*, 0µg/ml Cry5B | 0.972 | 0.015 | 0.959 | 0.019 | 0.013 |  |  | 0.9793 | ns |
| wild type, 5µg/ml Cry5B | *unc-31(e928)*, 5µg/ml Cry5B | 0.784 | 0.028 | 0.648 | 0.121 | 0.136 |  |  | 0.4908 | ns |
| wild type, 10µg/ml Cry5B | *unc-31(e928)*, 10µg/ml Cry5B | 0.678 | 0.036 | 0.314 | 0.099 | 0.363 |  |  | 0.0239 | * |
| wild type, 20µg/ml Cry5B | *unc-31(e928)*, 20µg/ml Cry5B | 0.288 | 0.072 | 0.044 | 0.023 | 0.244 |  |  | 0.0426 | * |
| wild type, 0µg/ml Cry5B | *unc-31(e928)*; neuronal rescue, 0µg/ml Cry5B | 0.972 | 0.015 | 0.982 | 0.018 | 0.011 |  |  | 0.9884 | ns |
| wild type, 5µg/ml Cry5B | *unc-31(e928)*; neuronal rescue, 5µg/ml Cry5B | 0.784 | 0.028 | 0.708 | 0.039 | 0.076 |  |  | 0.8260 | ns |
| wild type, 10µg/ml Cry5B | *unc-31(e928)*; neuronal rescue, 10µg/ml Cry5B | 0.678 | 0.036 | 0.497 | 0.072 | 0.181 |  |  | 0.2840 | ns |
| wild type, 20µg/ml Cry5B | *unc-31(e928)*; neuronal rescue, 20µg/ml Cry5B | 0.288 | 0.072 | 0.270 | 0.084 | 0.017 |  |  | 0.9945 | ns |
| wild type, 0µg/ml Cry5B | *egl-21(n476)*, 0µg/ml Cry5B | 0.972 | 0.015 | 0.883 | 0.050 | 0.088 |  |  | 0.1505 | ns |
| wild type, 5µg/ml Cry5B | *egl-21(n476)*, 5µg/ml Cry5B | 0.784 | 0.028 | 0.408 | 0.082 | 0.377 |  |  | 0.0210 | * |
| wild type, 10µg/ml Cry5B | *egl-21(n476)*, 10µg/ml Cry5B | 0.678 | 0.036 | 0.202 | 0.081 | 0.476 |  |  | 0.0056 | ** |
| wild type, 20µg/ml Cry5B | *egl-21(n476)*, 20µg/ml Cry5B | 0.288 | 0.072 | 0.090 | 0.015 | 0.197 |  |  | 0.0996 | ns |
| *unc-31(e928)*, 0µg/ml Cry5B | *unc-31(e928)*; neuronal rescue, 0µg/ml Cry5B | 0.942 | 0.015 | 0.982 | 0.018 | 0.023 |  |  | 0.8983 | ns |
| *unc-31(e928)*, 5µg/ml Cry5B | *unc-31(e928)*; neuronal rescue, 5µg/ml Cry5B | 0.696 | 0.164 | 0.708 | 0.039 | 0.060 |  |  | 0.9740 | ns |
| *unc-31(e928)*, 10µg/ml Cry5B | *unc-31(e928)*; neuronal rescue, 10µg/ml Cry5B | 0.417 | 0.160 | 0.497 | 0.072 | 0.182 |  |  | 0.3112 | ns |
| *unc-31(e928)*, 20µg/ml Cry5B | *unc-31(e928)*; neuronal rescue, 20µg/ml Cry5B | 0.097 | 0.063 | 0.270 | 0.084 | 0.227 |  |  | 0.0567 | ns |
| *Fig. S1A - fractions feeding after transfer to Cry21A* | | | | | | | | | | |
| 1min, control | 1min, Cry21A | 0.867 | 0.000 | 0.844 | 0.022 | 0.022 | -0.073 | 0.118 | 0.4226 | ns |
| 2min, control | 2min, Cry21A | 0.867 | 0.000 | 0.822 | 0.022 | 0.044 | -0.051 | 0.140 | 0.1835 | ns |
| 3min, control | 3min, Cry21A | 0.867 | 0.000 | 0.778 | 0.059 | 0.089 | -0.164 | 0.342 | 0.2697 | ns |
| 4min, control | 4min, Cry21A | 0.844 | 0.022 | 0.711 | 0.044 | 0.133 | -0.027 | 0.293 | 0.0765 | ns |
| 5min, control | 5min, Cry21A | 0.844 | 0.022 | 0.444 | 0.044 | 0.400 | 0.560 | 0.240 | 0.0043 | ** |
| 6min, control | 6min, Cry21A | 0.844 | 0.022 | 0.222 | 0.089 | 0.622 | 0.977 | 0.267 | 0.0153 | * |
| 7min, control | 7min, Cry21A | 0.844 | 0.022 | 0.000 | 0.000 | 0.844 | 0.749 | 0.940 | 0.0007 | *** |
| 8min, control | 8min, Cry21A | 0.844 | 0.022 | 0.000 | 0.000 | 0.844 | 0.749 | 0.940 | 0.0007 | *** |
| 9min, control | 9min, Cry21A | 0.844 | 0.022 | 0.000 | 0.000 | 0.844 | 0.749 | 0.940 | 0.0007 | *** |
| 10min, control | 10min, Cry21A | 0.844 | 0.022 | 0.000 | 0.000 | 0.844 | 0.749 | 0.940 | 0.0007 | *** |
| *Figure S1B - fractions feeding after transfer* | | | | | | | | | | |
| t = 0 | t = 1 min | 0.822 | 0.022 | 0.822 | 0.044 | 0.000 | -0.160 | 0.160 | 1.0000 | ns |
| t = 0 | t = 2 min | 0.822 | 0.022 | 0.822 | 0.044 | 0.000 | -0.160 | 0.160 | 1.0000 | ns |
| t = 0 | t = 3 min | 0.822 | 0.022 | 0.844 | 0.022 | 0.022 | -0.065 | 0.109 | 0.5185 | ns |
| t = 0 | t = 4 min | 0.822 | 0.022 | 0.844 | 0.022 | 0.022 | -0.065 | 0.109 | 0.5185 | ns |
| t = 0 | t = 5 min | 0.822 | 0.022 | 0.844 | 0.022 | 0.022 | -0.065 | 0.109 | 0.5185 | ns |
| t = 0 | t = 6 min | 0.822 | 0.022 | 0.844 | 0.022 | 0.022 | -0.065 | 0.109 | 0.5185 | ns |
| t = 0 | t = 7 min | 0.822 | 0.022 | 0.822 | 0.022 | 0.000 | -0.087 | 0.087 | 1.0000 | ns |
| t = 0 | t = 8 min | 0.822 | 0.022 | 0.822 | 0.022 | 0.000 | -0.087 | 0.087 | 1.0000 | ns |
| t = 0 | t = 9 min | 0.822 | 0.022 | 0.822 | 0.022 | 0.000 | -0.087 | 0.087 | 1.0000 | ns |
| t = 0 | t = 10 min | 0.822 | 0.022 | 0.844 | 0.022 | 0.022 | -0.065 | 0.109 | 0.5185 | ns |
| t = 0 | t = 0.5 hr | 0.822 | 0.022 | 0.844 | 0.022 | 0.022 | -0.065 | 0.109 | 0.5185 | ns |
| t = 0 | t = 2 hr | 0.822 | 0.022 | 0.800 | 0.000 | 0.022 | -0.118 | 0.073 | 0.4226 | ns |
| t = 0 | t = 24 hr | 0.822 | 0.022 | 0.778 | 0.022 | 0.044 | -0.132 | 0.043 | 0.2302 | ns |
| *Figure S1C - fractions feeding after transfer to 24hr-old Cry5B* | | | | | | | | | | |
| 10min, control | 10min, Cry5B | 0.790 | 0.063 | 0.000 | 0.000 | 0.790 | 0.517 | 1.063 | 0.0064 | ** |
| 30min, control | 30min, Cry5B | 0.823 | 0.057 | 0.000 | 0.000 | 0.823 | 0.573 | 1.070 | 0.0048 | ** |
| *Fig. S2A - fractions feeding after transfer to Cry21A* | | | | | | | | | | |
| wild type, control, 10min | wild type, Cry21A, 10min | 0.844 | 0.022 | 0.000 | 0.000 | 0.844 |  |  | <0.0001 | *** |
| *goa-1(sa734)*, control, 10min | *goa-1(sa734)*, Cry21A, 10min | 0.778 | 0.022 | 0.778 | 0.022 | 0.000 |  |  | 1.0000 | ns |
| wild type, control, 10min | *goa-1(sa734)*, control, 10min | 0.844 | 0.022 | 0.778 | 0.022 | 0.067 |  |  | 0.1012 | ns |
| wild type, Cry21A, 10min | *goa-1(sa734)*, Cry21A, 10min | 0.000 | 0.000 | 0.778 | 0.022 | 0.778 |  |  | <0.0001 | *** |
| wild type, control, 0.5hr | wild type, Cry21A, 0.5hr | 0.756 | 0.022 | 0.000 | 0.000 | 0.756 |  |  | <0.0001 | *** |
| *goa-1(sa734)*, control, 0.5hr | *goa-1(sa734)*, Cry21A, 0.5hr | 0.733 | 0.000 | 0.733 | 0.038 | 0.000 |  |  | 1.0000 | ns |
| wild type, control, 0.5hr | *goa-1(sa734)*, control, 0.5hr | 0.756 | 0.022 | 0.733 | 0.000 | 0.022 |  |  | 0.3739 | ns |
| wild type, Cry21A, 0.5hr | *goa-1(sa734)*, Cry21A, 0.5hr | 0.000 | 0.000 | 0.733 | 0.038 | 0.733 |  |  | <0.0001 | *** |
| wild type, control, 2hr | wild type, Cry21A, 2hr | 0.756 | 0.022 | 0.000 | 0.000 | 0.756 |  |  | <0.0001 | *** |
| *goa-1(sa734)*, control, 2hr | *goa-1(sa734)*, Cry21A, 2hr | 0.711 | 0.022 | 0.689 | 0.044 | 0.022 |  |  | 0.6779 | ns |
| wild type, control, 2hr | *goa-1(sa734)*, control, 2hr | 0.756 | 0.022 | 0.711 | 0.022 | 0.044 |  |  | 0.2302 | ns |
| wild type, Cry21A, 2hr | *goa-1(sa734)*, Cry21A, 2hr | 0.000 | 0.000 | 0.689 | 0.044 | 0.689 |  |  | 0.0001 | *** |
| *Fig. S2B - fractions feeding after transfer to Cry5B* | | | | | | | | | | |
| wild type, control, 10min | wild type, Cry5B, 10min | 0.630 | 0.084 | 0.000 | 0.000 | 0.630 |  |  | 0.0017 | ** |
| *goa-1(ep275)*, control, 10min | *goa-1(ep275)*, Cry5B, 10min | 0.712 | 0.038 | 0.798 | 0.060 | 0.086 |  |  | 0.2914 | ns |
| wild type, control, 10min | *goa-1(ep275)*, control, 10min | 0.630 | 0.084 | 0.712 | 0.038 | 0.083 |  |  | 0.4228 | ns |
| wild type, Cry5B, 10min | *goa-1(ep275)*, Cry5B, 10min | 0.000 | 0.000 | 0.798 | 0.060 | 0.798 |  |  | 0.0002 | *** |
| wild type, control, 0.5hr | wild type, Cry5B, 0.5hr | 0.640 | 0.034 | 0.000 | 0.000 | 0.640 |  |  | <0.0001 | *** |
| *goa-1(ep275)*, control, 0.5hr | *goa-1(ep275)*, Cry5B, 0.5hr | 0.614 | 0.086 | 0.706 | 0.103 | 0.092 |  |  | 0.5293 | ns |
| wild type, control, 0.5hr | *goa-1(ep275)*, control, 0.5hr | 0.640 | 0.034 | 0.614 | 0.086 | 0.026 |  |  | 0.7904 | ns |
| wild type, Cry5B, 0.5hr | *goa-1(ep275)*, Cry5B, 0.5hr | 0.000 | 0.000 | 0.706 | 0.103 | 0.706 |  |  | 0.0024 | ** |
| wild type, control, 2hr | wild type, Cry5B, 2hr | 0.655 | 0.038 | 0.000 | 0.000 | 0.655 |  |  | <0.0001 | *** |
| *goa-1(ep275)*, control, 2hr | *goa-1(ep275)*, Cry5B, 2hr | 0.573 | 0.079 | 0.521 | 0.098 | 0.052 |  |  | 0.6997 | ns |
| wild type, control, 2hr | *goa-1(ep275)*, control, 2hr | 0.655 | 0.038 | 0.573 | 0.079 | 0.081 |  |  | 0.4048 | ns |
| wild type, Cry5B, 2hr | *goa-1(ep275)*, Cry5B, 2hr | 0.000 | 0.000 | 0.521 | 0.098 | 0.521 |  |  | 0.0059 | ** |
| *Fig. S2C - pumping rates 30 min after transfer* | | | | | | | | | | |
| wild type, non-transferred | *goa-1(n1134) egl-30(n686)*, non-transferred | 251 | 244-262 | 166 | 0-184.5 | 85 |  |  | <0.0001 | *** |
| wild type, non-transferred | *goa-1(sa734)*, non-transferred | 251 | 244-262 | 228 | 218-234 | 23 |  |  | <0.0001 | *** |
| wild type, non-transferred | *eat-16(ce71)*, non-transferred | 251 | 244-262 | 104 | 1-175 | 147 |  |  | <0.0001 | *** |
| wild type, non-transferred | *egl-30(n686)*, non-transferred | 251 | 244-262 | 181 | 67.5-204 | 70 |  |  | <0.0001 | *** |
| wild type, non-transferred | *egl-10(n692)*, non-transferred | 251 | 244-262 | 222 | 79-233 | 29 |  |  | <0.0001 | *** |
| wild type, non-transferred | *goa-1(n1134)*, non-transferred | 251 | 244-262 | 238 | 166-244 | 13 |  |  | 0.0003 | *** |
| wild type, non-transferred | *egl-10(xs)*, non-transferred | 251 | 244-262 | 208 | 0-228 | 43 |  |  | <0.0001 | *** |
| wild type, non-transferred | wild type, 0% | 251 | 244-262 | 256 | 0-269.5 | 5 |  |  | 0.6359 | ns |
| *goa-1(n1134) egl-30(n686)*, non-transferred | *goa-1(n1134) egl-30(n686)*, 0% | 166 | 0-184.5 | 126 | 0-178 | 40 |  |  | 0.1583 | ns |
| *goa-1(sa734)*, non-transferred | *goa-1(sa734)*, 0% | 228 | 218-234 | 219 | 170-229.5 | 9 |  |  | 0.1173 | ns |
| *eat-16(ce71)*, non-transferred | *eat-16(ce71)*, 0% | 104 | 1-175 | 92 | 3-146 | 12 |  |  | 0.7716 | ns |
| *egl-30(n686)*, non-transferred | *egl-30(n686)*, 0% | 181 | 67.5-204 | 164 | 0-203 | 17 |  |  | 0.4581 | ns |
| *egl-10(n692)*, non-transferred | *egl-10(n692)*, 0% | 222 | 79-233 | 216 | 0-238 | 6 |  |  | 0.9621 | ns |
| *goa-1(n1134)*, non-transferred | *goa-1(n1134)*, 0% | 238 | 166-244 | 237 | 69.5-243.5 | 1 |  |  | 0.7569 | ns |
| *egl-10(xs)*, non-transferred | *egl-10(xs)*, 0% | 208 | 0-228 | 216 | 0-232 | 8 |  |  | 0.9825 | ns |

^1^Conditions A and B are the two treatments that are compared, with corresponding mean and standard error of the mean (S.E.M.). For Fig. 2D and S2B, this shows the median and interquartile range instead.

^2^Difference between the two means A and B.

^3^Statistical tests used for calculation of upper and lower confidence limits (CL), and p-value described in Materials and Methods. Where required to allow statistical analysis a mean or S.E.M. of 0.001 was used instead of 0 to calculate p.

^4^ns: not significant, *: p<0.05, **: p<0.01, ***: p<0.001
