# Supplementary material for: Neuronal Goα and CAPS Regulate Behavioral and Immune Responses to Bacterial Pore-Forming Toxins
Source: PLoS One. 2013 Jan 17;8(1):e54528. doi: 10.1371/journal.pone.0054528 (PMC3547950; doi:10.1371/journal.pone.0054528)
Supplement: Table S3 — C. elegans strains used in this study. (DOCX) [file pone.0054528.s006.docx]

**Table S3. *C. elegans* strains used in this study**

| **strain** | **genotype** | **figure/table** |
| --- | --- | --- |
| N2 | wild type | all |
| KG524 | *gsa-1(ce94)* | Table S2 |
| KG522 | *acy-1(md1756)* | Table S2 |
| KG744 | *pde-4(ce268)* | Table S2 |
| KG532 | *kin-2(ce179)* | Table S2 |
| DG1856 | *goa-1(sa734)* | Figure 2A, C, D, E, 3A, B, C, S2; Table S2 |
| MT2426^1^ | *goa-1(n1134)* | Figure 2E, 3B, C, S2; Table S2 |
| MT1434 | *egl-30(n686)* | Figure 2E, 3B, S2; Table S2 |
| CG21^2^ | *egl-30(tg26); him-5(e1490)* | Table S2 |
| NM1380 | *egl-30(js126)* | Table S2 |
| AQ812 | *goa-1(n1134) egl-30(n686)* | Figure 2E, 3B, S2; Table S2 |
| KG571 | *eat-16(ce71)* | Figure 2E, 3A, B, C, S2; Table S2 |
| MT1443 | *egl-10(n692)* | Figure 2E, 3B, S2; Table S2 |
| MT8190^3^ | *lin-15B(n765);nIs51(pEK1[lin-15+];pMK21[egl-10+])* | Figure 2E, 3B, C, S2; Table S2 |
| RM2209 | *ric-8(md1909)* | Table S2 |
| KP1097 | *dgk-1(nu62)* | Table S2 |
| MT1083 | *egl-8(n488)* | Table S2 |
| MT7929 | *unc-13(e51)* | Table S2 |
| GR1321 | *tph-1(mg280)* | Table S2 |
| CB928 | *unc-31(e928)* | Figure 4A, B; Table S2 |
| KG835 | *unc-31(e928);ceEx117(rab-3P::unc-31(+) cDNA)^4^* | Figure 4B; Table S2 |
| KP2018 | *egl-21(n476)* | Figure 4A, B; Table S2 |
| VC461 | *egl-3(gk238)* | Table S2 |
| KG855^1^ | *goa-1(sa734);unc-31(e928)* | Table S2 |
| PS1493^5^ | *dpy-20(e1362);syIs9(goa-1P::goa-1(Q205L))* | Figure 3B |
| CE1051^6^ | *goa-1(ep275)* | Figure 2B, S3 |

Unless otherwise noted, strains were outcrossed at least four times and obtained from the Caenorhabditis Genetics Center (CGC).

^1^Unknown how often outcrossed.

^2^Outcrossed 3x. Only hermaphrodites were tested.

^3^*egl-10* is overexpressed in this strain using 4,000 base pairs of 5’ flanking sequence, and we therefore assume localization of its expression is the same as for native *egl-10* (Koelle MR, Horvitz HR (1996) Cell 84: 115-125).

^4^Only animals carrying transgene were tested.

^5^Outcrossed ≥2x.

^6^Outrossed 1x.
